# Musculoskeletal disorders and pain in agricultural workers in Low- and Middle-Income Countries: a systematic review and meta-analysis

**DOI:** 10.1007/s00296-023-05500-5

**Published:** 2023-11-24

**Authors:** Mrithula Shivakumar, Victoria Welsh, Ram Bajpai, Toby Helliwell, Christian Mallen, Michelle Robinson, Thomas Shepherd

**Affiliations:** Keele University, Keele, David Weatherall Building, Newcastle-under-Lyme, ST5 5BG UK

**Keywords:** Musculoskeletal diseases, Low back pain, Agricultural workers, Farmers, Low- and Middle-Income Countries

## Abstract

Agricultural workers constitute two-thirds of the population of Low- and Middle-Income Countries (LMIC) and are at increased risk of developing musculoskeletal disorders (MSD) due to high-risk activities. This systematic review and meta-analysis aim to synthesise the prevalence, predictors, and outcomes of musculoskeletal pain amongst agricultural workers to identify priority areas for prevention and development of early interventions. This systematic review and meta-analysis included Studies published from the inception of global electronic databases until 30 September 2022 were included. Prevalence estimates for MSDs among agricultural workers aged over 18 years in LMIC were extracted. Narrative synthesis summarized study findings and pooled estimates for 12-month pain prevalence were calculated. 7502 potential studies were identified. 64 studies (68,684 participants from 23 countries) were included in the systematic review; 33 studies were included in the meta-analysis. Low back pain was the most widely investigated symptom. The 12-month pooled prevalence of low back pain was highest in Africa [61.96% (45.69–76.22)] compared to Asia [54.16% (47.76–60.50)] and South/Central America [28.52%(10.91–50.33)]. Narrative synthesis found associations between MSDs, particular activities including heavy lifting and repetitive movements, and outcomes including reduced productivity. MSDs are common in agriculture workers in LMIC. Global prevalence of low back pain in farmers, particularly in Africa, is greater than in previously reported global prevalence in the general population. This may be attributed to environmental factors and high-risk activities which could be targeted for prevention and early intervention strategies to support individuals, prevent disability, and reduce loss of productivity.

**Supplementary Information:**

The online version contains supplementary material available at 10.1007/s00296-023-05500-5.

## Introduction

Musculoskeletal diseases (MSD), defined by the World Health Organisation as disorders affecting the locomotor system, including disorders of the muscles, bones, joints and, connective tissues [1], are associated with a high disease burden globally, and commonly present with musculoskeletal pain. Low back pain was identified as the ninth leading cause of Disability Adjusted Life Years (DALY) in all ages and fourth leading cause in the working population of ages 25–49 [2]. MSD such as low back and other regional pain can detrimentally affect livelihood with an impact on daily activities, social relationships, and employment [3].

Evidence from high-income countries highlights the key role of occupation in the development and maintenance of musculoskeletal disorder and pain-related conditions [4]. To date, evidence from Low- and Middle-Income Countries (LMIC) has not been synthesised despite the high rates of work in physically demanding occupations such as agriculture, which currently contributes to 23.34% to GDP for low-income, and 8.83% for middle-income countries [5]. Without knowing the prevalence of pain in this population it is not possible to design and implement preventative strategies to support agricultural workers in their musculoskeletal health to ensure continued productivity and facilitate economic growth.

Although improvements have been made, the quality of care for people with musculoskeletal disorders is often inadequate across LMIC and particularly in vulnerable groups [6]. Limited resources in LMIC are often channelled towards communicable diseases, such as HIV and malaria. Understanding the musculoskeletal health of agricultural workers in LMIC will provide the evidence needed to support allocation of resources to optimise the health and well-being of this population including potential low-cost solutions for improving access to healthcare and outcomes for those most in need [7].

This systematic review and meta-analysis aims to identify the prevalence, predictors, and outcomes of musculoskeletal disorders, including pain, amongst agricultural workers to identify priority areas for prevention and development of early interventions.

## Methods

We adhered to the Preferred Reporting Items for Systematic Reviews and Meta-Analyses (PRISMA) 2020 statement for reporting this systematic review [8]. The study was registered with PROSPERO (CRD42018094183).

### Search strategy

This systematic review and meta-analysis include publications including agricultural workers in LMIC aged 18 years and over. All prevalence durations, body sites and phenotypes of musculoskeletal disease and chronic pain were included. All methods for measurement of MSD and pain prevalence were included, for example self-completed survey or clinical examination. Studies were excluded if it was not possible to obtain a full text, or if it was not possible to separate populations of farmers or children.

Agricultural work was defined as all types of farming including rearing livestock, horticulture, fruit growing, seed growing, maintaining woodlands, pastures, meadows, market gardens and nursery grounds and reedbeds as specified in the Agricultural Act 1947. Fishing was excluded as it did not meet the requirements of this definition. All languages were included, and translators were identified for papers published in Portuguese, Spanish, and Farsi. Remaining texts were translated using Google Translate (Google LLC, Mountain View, USA). Observational quantitative studies. The search was conducted in relevant health-related databases from inception to September 2020 including Medline, Embase, PsycINFO, IMSEAR, LILACS, AIM, WPRIM, IMEMR, Pakmedinet, BanglaJOL, Sri Lanka journals online, WHO library database. An updated identical search was conducted for October 2020–September 2022, with the exception of IndMed which was no longer available.

For MEDLINE, Embase, PsychInfo, the search strategy included the exploded Medical Subject Heading (MeSH) terms and ‘multiple places’ command (.mp) for words relating to MSD, agriculture, and LMICs were combined using AND function (Supplementary Information: Table 1). All 137 countries and name-variants listed by the World Bank in 2018 when the search. The most recent search update in 2022 included new name-variants for countries listed by the World Bank in 2022. Countries such as Nauru, Panama, and Romania are now in high-income category as of 2022. Palau was included in the update as it a middle-income country as of 2022. Due to limitations in the search facility for other sources, keywords searched included ‘pain’ or ‘musculoskeletal’ AND ‘agricultur*’ or ‘farm’. The WHO global databases (IMSEAR, LILACS, AIM, WPRIM and IMEMR) were searched using the terms “pain” or “musculoskeletal” AND “agricultur* or “farming” or “farmer*”.

### Study selection

Study authors were contacted if full-text versions of conference abstracts were unavailable, or if unpublished data were required to calculate prevalence. Titles were screened using Covidence for de-duplication and screening. Two reviewers (from VW, MS, MR) screened titles and abstracts and then full texts. Any duplicates not identified by Covidence were removed by screeners and any disagreements or doubts were clarified with the whole team.

### Main outcome variables

The primary outcome was the prevalence of musculoskeletal disorders in agricultural workers in LMIC over 18 years old. Secondary outcomes were to identify risk factors associated with MSK disorders and burdens of MSK disorders in agricultural workers in LMIC.

Reviewers reported prevalence estimates, associated factors and burdens associated with the reported MSK disorders. Data was extracted from manuscripts and entered in a piloted Excel spreadsheet. Verification of data entry was undertaken by three reviewers (MS, VW, MR). Data collected included, where available, study identification (author name, year of publication), funding source, type of study, type of publication (journal article conference abstract), study design, setting and source of study population (primary care/ community; country or origin), selection criteria, study sample size, age distribution (range, mean, standard deviation), sex distribution (counts and proportions), data collection method (e.g. self-report questionnaire, interview), disorder description, classification and measurement, description of other measured characteristics (for example other demographic information), outcome (pain prevalence), associated factors and disorder burdens (which predictors have been explored, their measures and effect estimates (risk estimate, 95% CI and *p* value), date of data extraction and data extractor.

### Risk of bias assessment

A quality appraisal tool specifically designed for prevalence studies was used to assess study quality [9]. The tool scores external validity (four items) and internal validity (six items) as high and low risk and asks for an overall risk of bias categorised as low, moderate, or high. Quality assessment was carried out by three reviewers (VW, MS, MR) independently for all included articles. Discrepancies in quality appraisal scores were settled with a third reviewer. Data extraction was undertaken by three reviewers (MS, VW, MR), to verify accuracy of extraction.

### Data synthesis

Study characteristics were summarised and, for pain phenotypes and duration that were not amenable for meta-analysis, narrative synthesis was completed. Meta-analysis was undertaken for phenotypes and reported pain durations where sufficient numbers of studies were available, which were 12-month prevalence estimates for low back pain and pain and different body sites. Pain prevalence was calculated as the ratio between the total number of people with musculoskeletal pain over the total study population and presented as the number of cases per 100 population. For individual studies, 95% confidence intervals (CI) were determined using the Wilson method from the reported crude estimates and population denominators [10]. Random-effects model was used to produce a pooled prevalence estimate and DerSimonian and Laird method were employed to calculate its 95% CI [11]. Between studies heterogeneity was assessed using the *χ*^2^-based Cochran’s *Q* test and tau-squared estimate. The proportion of variability in prevalence due to between-study heterogeneity was summarised using *I*-squared (*I*^2^) statistics. A prediction interval for the random-effects distribution was also calculated to understand the possible range of musculoskeletal pain prevalence if a new study is conducted in low- and middle-income countries as suggested by Higgins and Thompson [12]. Publication bias was assessed by funnel plot if ten or more studies were available, and its asymmetry was tested by Egger’s linear regression method. A *p* value of < 0.05 was considered statistically significant for the effect of study-level covariates on the estimated prevalence. All statistical analyses were conducted on Stata software version 17.0 (StataCorp, College Station, Texas, USA) using “metan” package.

No external funding was associated with this study.

## Results

**Fig. 1:**
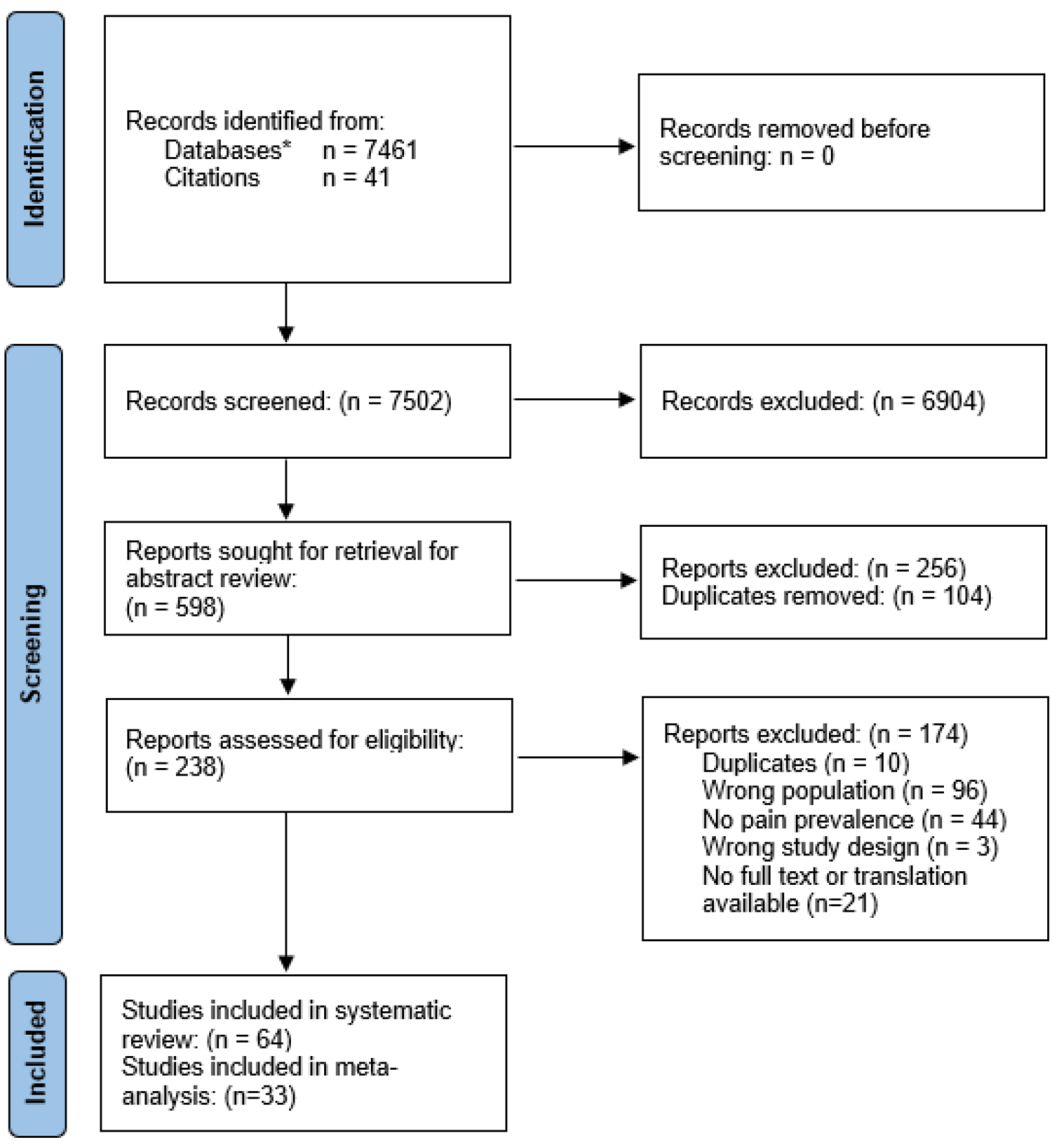
PRISMA flow diagram of the study selection process

Seven thousand four hundred sixty-one eligible studies were identified through database search and 41 identified through hand-searching reference lists. 64 studies were included in the systematic review (Supplementary Information: Table 2) [13–76], with 33 studies included in the meta-analysis (Fig. 1) [15, 17–21, 24, 25, 31–33, 37, 38, 41, 48–52, 54, 56, 59–64, 68–71, 75, 76].

### Study characteristics

**Fig. 2:**
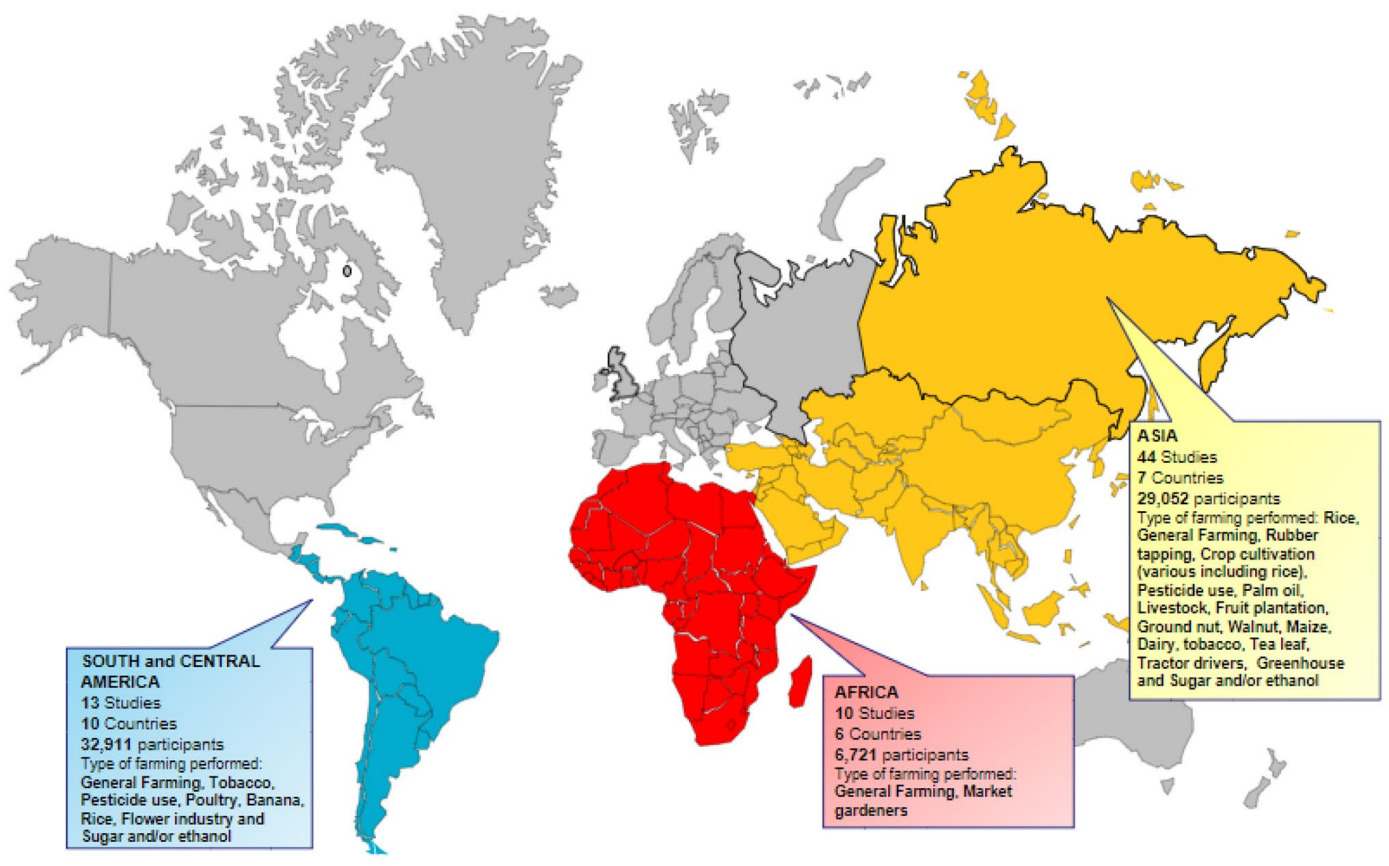
World map denoting included studies (*n* = 64) and number of participants

The included studies were conducted from 1998 to 2022. A total of 68,684 participants from 23 countries were included (Fig. 2), with an additional unknown number of farmers reported as a subgroup in one study by Bihari et al. [27]. Ten studies were conducted in Africa, 44 in Asia, 13 in South and Central America. The Nordic Musculoskeletal Questionnaire and variations translated in local languages were the mostly used tool (31 studies) for pain measurement [77]. A summary of the study characteristics can be found in Table 1.

**Table 1:** A summary of study characteristics

Geographical region	*n*
Africa	10
Asia	45
South/Central America	9
Type of farming*	
General farming/not specified	19
Flower	1
Crop cultivation (various including rice, maize, ground nut, walnut)	16
Livestock, poultry, dairy and crop cultivation	4
Rubber tapping	5
Tobacco	5
Sugar/ethanol	3
Palm oil	3
Fruit farming	3
Tea	2
Greenhouse	3
Market garden	1
Tractor driving only (no other farming activity)	1
Pain prevalence period*	
Point-prevalence	17
Last 24 h	3
Last 7 days	12
Last 2 weeks	1
Last 1 month	3
Last 3 months	5
Last 6 months	1
Last 12 months	35
Lifetime	3
Unclear/not specified	9
Methods for obtaining prevalence	
Standardised Nordic Questionnaire/Modified Nordic Questionnaire	31
Other questionnaire	19
Clinical examination and questionnaire	5
Unclear/not specified	8
Total participants (farmers)	
1–50 participants	6
51–100 participants	5
101–500 participants	36
501–1000 participants	5
Over 1000 participants	12

Risk of bias	Cross-sectional	Case control	Cohort	Case report	Total
Low	17	0	0	0	17
Medium	36	2	1	1	40
High	7	0	0	0	7

*Some studies included in the total more than once as they reported multiple types of farming and pain prevalence periods

### 12-month pooled prevalence estimates for low back pain

**Fig. 3:**
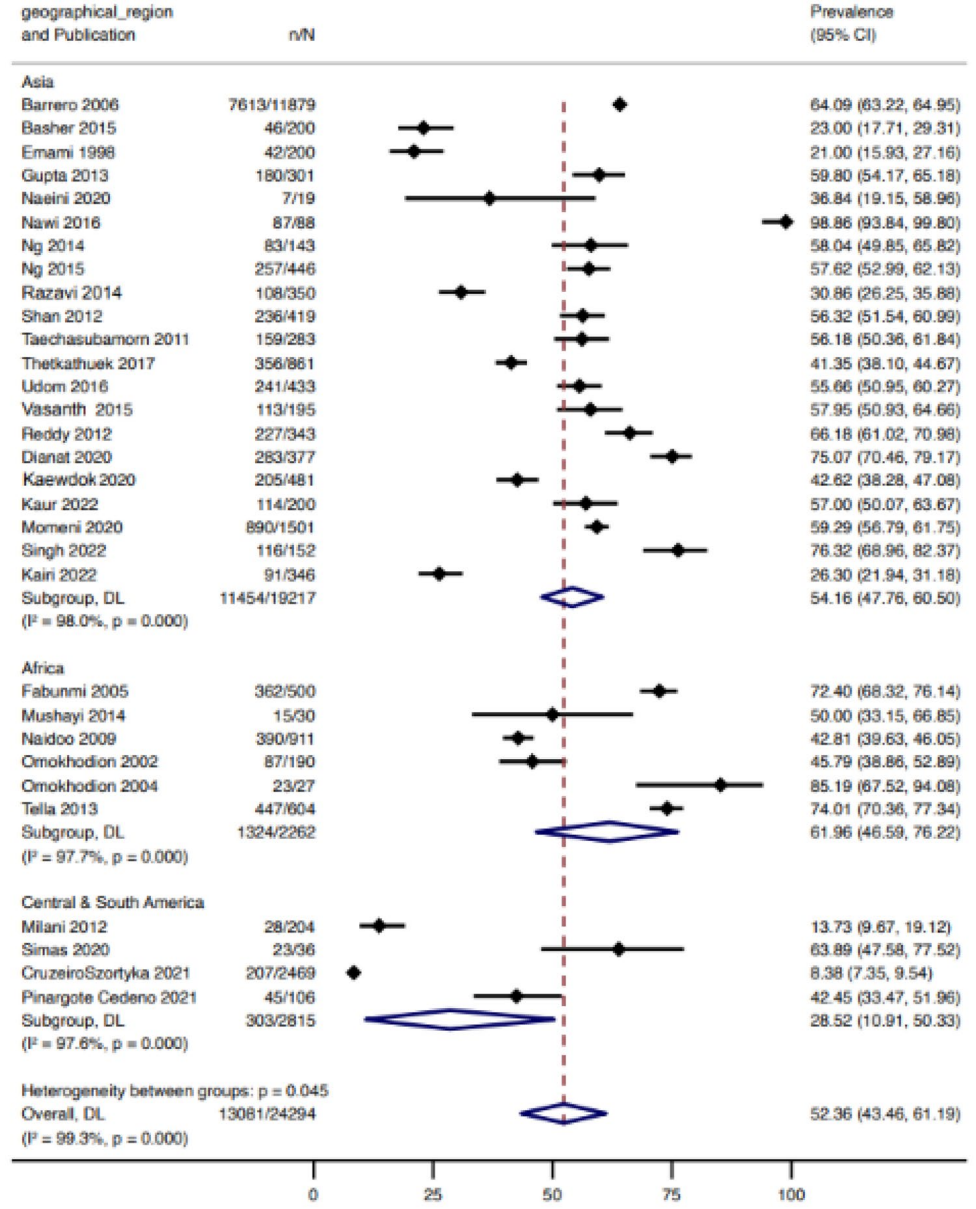
12-month pooled prevalence estimate of low back pain in agricultural workers in LMIC

**Fig. 4:**
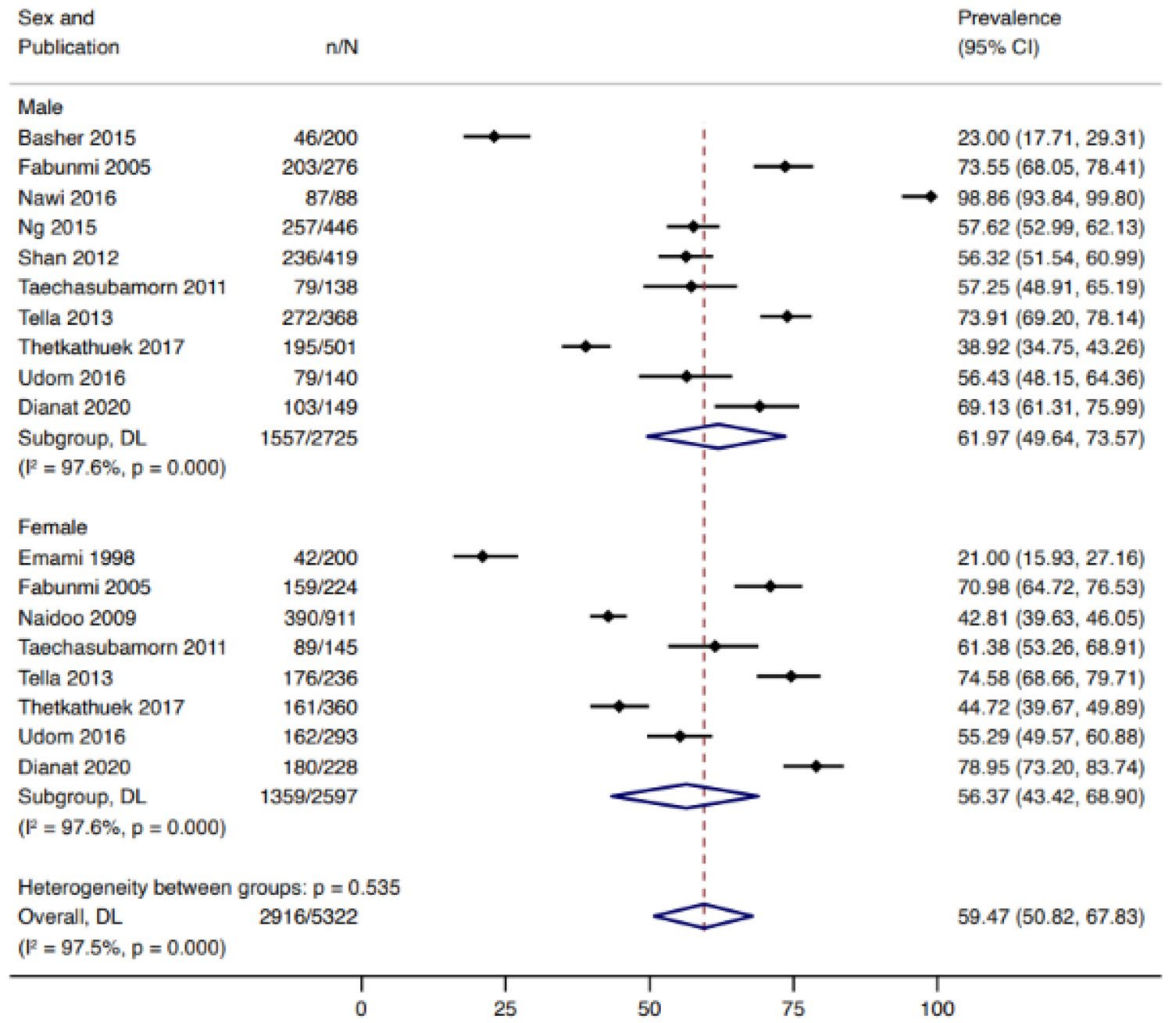
12-month pooled prevalence estimate of low back pain prevalence in male and female agricultural workers in LMICs

LBP was the most widely investigated musculoskeletal disorder with prevalence estimates reported in 47 studies. Of these, 33 studies reported 12-month pain prevalence of low back pain (presented in Fig. 3). The 12-month prevalence of low back pain was highest in African countries with estimates of 61.96% (95% CI 45.69–76.22) followed by Asia [54.16% (95% CI 47.76–60.50)] and South and Central America [28.52% (95% CI 10.91–50.33)]. Funnel plot visually indicated some form of publication bias as the study distribution was not symmetric (Supplementary Information 3). However, we did not observe statistical evidence for the small-study effect (*p* = 0.644). When stratified by sex, males reported a higher prevalence of low back pain (61.97%, 95% CI 49.64–73.57 from 11 studies) compared to females (56.37%, 95% CI 43.42–68.90 from 9 studies) (Fig. 4).

### 12-month pooled prevalence estimates according to body site

**Fig. 5:**
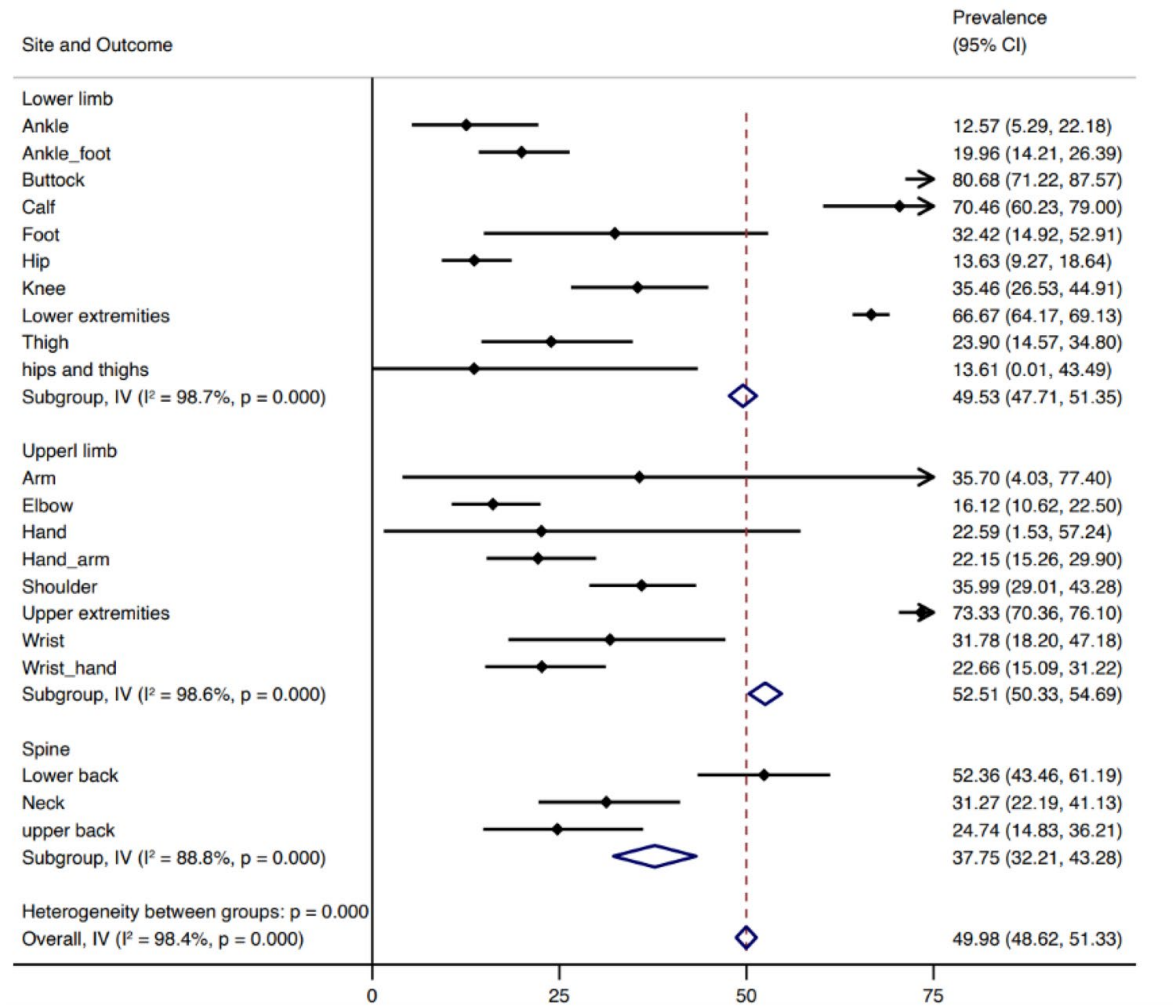
12-month pooled prevalence estimates for musculoskeletal pain according to body site

The 12-month reported pain prevalence varied according to body site/pain location, as demonstrated in Fig. 5. For example, the 12-month prevalence of lower limb pain was 49.53% (47.71, 51.35), upper limb pain was 52.51 (50.33, 54.69), spinal pain was 37.75 (32.21, 43.28). Substantial overall statistical heterogeneity was observed across studies within and between countries.

### Associated factors with reported pain: narrative synthesis

Supplementary Information 4 contains a table detailing study characteristics and associations with reported pain for studies included in the narrative synthesis that investigated these aspects; the commonly measured variables are presented here. The association of MSDs and musculoskeletal pain with age, sex, and BMI was not consistent across studies. Increasing age was significantly associated with greater numbers of farmers reporting musculoskeletal pain in 14 studies, though one study did not find an association with age and one study suggested a healthy cohort effect, where pain was greatest in ages 39–48 compared to age 49 and older [59, 61]. Three studies reported no significant relationship between sex and MSDs; two studies found men reporting more MSDs (*p* < 0.05) and three studies found that women reported more symptoms than men (*p* < 0.05) [21, 36, 37, 60, 62]. Regarding BMI, one study found increasing BMI was associated with fewer symptoms (*p* < 0.05) [52], and two studies found increasing BMI was associated with more symptoms (*p* < 0.05) [63, 76]. Comorbidity was associated with increased MSD reporting (*p* < 0.05)[64]. No significant relationship was found with smoking tobacco or drinking alcohol [44]. One study found a significant association with smoking tobacco or ex-smokers only in females (*p* < 0.05) and not in males [68]. Comorbidity was associated with increased MSD reporting (*p* < 0.05)[64]. No significant relationship was found with smoking tobacco or drinking alcohol [44]. One study found a significant association with smoking tobacco or ex-smokers only in females (*p* < 0.05) and not in males [68]. Six studies measured the effect of education on MSD reporting; two studies found a non-significant relationship [13, 44], and lower educational status was associated with greater reporting of MSDs in three studies (*p* < 0.05) [22, 52, 63]. Two studies that measured the impact of income on MSD reporting found low income was associated with greater symptom reporting (*p* < 0.05) [22, 47]. Moderate and high levels of stress, regular stress, stress over future income, anxiety and disorders of the sleep–wake cycle were associated with MSDs in studies investigating the impact of stress (*p* < 0.05) [42, 44, 53, 72]. High perceived work fatigue was also associated with greater reporting of MSDs (*p* < 0.05) [36, 47]. Other associations with increased MSD reporting, were high gravidity, rural residence, no outreach health services, firewood cooking method and current breastfeeding [22].

Six studies measured the effect of education on MSD reporting; two studies found a non-significant relationship [13, 44], and lower educational status was associated with greater reporting of MSDs in three studies (*p* < 0.05) [22, 52, 63]. Two studies that measured the impact of income on MSD reporting found low income was associated with greater symptom reporting (*p* < 0.05) [22, 47]. Moderate and high levels of stress, regular stress, stress over future income, anxiety and disorders of the sleep–wake cycle were associated with MSDs in studies investigating the impact of stress (*p* < 0.05) [42, 44, 53, 72]. High perceived work fatigue was also associated with greater reporting of MSDs (*p* < 0.05) [36, 47]. Other associations with increased MSD reporting, were high gravidity, rural residence, no outreach health services, firewood cooking method and current breastfeeding [22].

Longer working hours and longer duration of employment were significantly associated with increased reporting of MSDs [13, 18, 25, 31, 62, 64, 76], though one study found that working for more than 5 days in a field was associated with less pain (instead of working in other non-field-based tasks) and one study did not find daily hours, nor farming experience, were significantly associated with MSDs [36, 42].

Farming practices that were significantly associated with increased MSDs (those with reported *p* < 0.05) were heavy physical exertion, strenuous manual labour and intensive farm work [22, 55], awkward postures including frequent kneeling or squatting [18, 28, 37, 53, 55, 59]; activities overhead, above eye level and below waist level, or below knee level [17, 18, 47, 63]; frequent lifting or carrying of loads > 5 kg [18, 22, 43, 47], walking with a back bend or restricted posture [17, 52], pulling or pushing heavy objects [17], collecting loose fruit [52], repetitive motions [53], forceful exertions, and static postures [59]. Reporting responsibility for a greater number of livestock and larger plantation areas was also significantly associated with increased MSD reporting (*p* < 0.05) [55, 62]. Driving a tractor was significantly associated with greater low back pain reporting and abnormal clinical examination of the knee compared with non-tractor driving farmers (*p* < 0.05) [45]. One study investigating pesticide use found that those reporting chest pain had statistically greater laboratory abnormalities in two of the four tests, whilst there was no associated muscle weakness [35]; the other study that measured pesticide use found increased reporting of MSDs with increased exposure to pesticides (*p* < 0.05) [63].

### Burdens associated with pain reporting: narrative synthesis

Supplementary Information 5 contains a table detailing pain and associated burdens for studies included in the narrative synthesis that investigated these aspects; the commonly measured variables are presented here. One study found 56.5% of workers reporting MSD symptoms found they impacted upon day-to-day activities [17]; upper back, knee, and wrist/hand pain (2.90%, 2.50%, 2.50% of those reporting pain respectively) were the most common sites to prevent normal activity [56]. Five studies investigated the impact of MSDs upon work; productivity was reduced and presenteeism was greater in those reporting symptoms compared to those reporting no MSD symptoms (*p* < 0.05) [51]. Other narratively reported outcomes included that more than half of farmers with low back pain reported difficulty carrying out their work and reported absence from work due to the pain [21], and shoulder pain was cited as a reason to change jobs, with one in five reporting difficulty doing their normal work [64]. Neck pain and low back pain were also cited reasons that caused difficulty carrying out usual farm work and 86% of farmers in one study temporarily stopped their work due to pain [25, 64]. Three-quarters of farmers in one study reported visiting a doctor due to pain (*p* < 0.05), and 99.5% of workers had seen a doctor for neck pain or low back pain in another, 7.4% of whom underwent hospitalisation. One study found that 23.1% of those reporting low back pain necessitated a hospital visit [21], and another study found that upper back and knee pain were the commonest pain sites to require a hospital visit (5.4% and 5.4% of all respondents respectively) [56]. Half of workers with pain attended a specialist, and a third attended the emergency room in one study [72]. The same study found that self-medicating, resting, massage, homemade remedies and prescribed drug therapies were used as pain management strategies. One study found low back pain was associated with suicidal ideation (*p* < 0.05) [69].

### Risk of bias assessment

There was a high degree of heterogeneity across the 64 included studies. Prevalence estimates varied from point prevalence, symptoms for 3, 4, 6 or 12 months, or lifetime prevalence. There was variation in the definition of MSK pain. 31 studies used the NMQ questionnaire (including variations of the NMQ) to define pain. 19 studies used other questionnaires such as the Cornell Musculoskeletal Discomfort questionnaire [78], or questionnaires piloted by the study authors. Five studies used clinical examination in addition to interview. Eight studies did not clearly specify methodology for measuring pain outcomes. Six studies reported MSDs as part of a study investigating pesticide use [14, 26, 35, 55, 63, 65]. Risk of bias assessment (detailed in Supplementary Information 6) scored 17 studies as low risk, 40 studies as moderate risk and seven studies as high risk of bias. Studies were judged to be high risk due to limited methodology information, absent information to enable representativeness of the study sample to be evaluated and omission of raw data presentation in the publication.

## Discussion

Musculoskeletal disorders, including pain, are commonly reported by agricultural workers in LMIC. Low back pain was the most investigated symptom across studies, with 52% of farmers reporting low back pain in the previous 12 months. The global prevalence of low back pain has previously been reported as 38.0% in a meta-analysis that synthesised all published estimates globally in the general population regardless of occupation [79]. Globally, 12-month pain prevalence in agricultural workers has been reported as 47.8% in a study including mostly high-income countries [80]. In LMIC, it has been previously reported that the general population has a low back pain prevalence of 21% [81]. Our findings demonstrate that agricultural workers have a higher prevalence of low back pain than the general population.

Low back pain prevalence estimates varied across geographical regions with Africa reporting greater 12-month pain prevalence [61.96% (95% CI 45.69–76.22)] as compared to South and Central America [28.52% (95% CI 10.91–50.33)]. A previous systematic review assessing musculoskeletal pain in the general population of LMIC in Africa found 12-month low back pain prevalence to be 57% [82]. Prevalence estimates of low back pain in Latin America based on epidemiological modelling has been reported to be 10.5% [83]. This may be due to variations in farming practices in these different regions as described in the studies or heterogeneity in the way in which pain is measured. For example, studies based in Africa included populations working in subsistence or small-scale farming which involves each individual performing a variety of tasks and high-risk activities (e.g. stooping, arms above head, heavy lifting, twisting and repetitive movements) rather than one specific type of farming activity (e.g. driving, threshing, sowing) [18, 22].

12-month pain prevalence was found to be similar in the upper limb, lower back, and lower limb as 52.51%, 52.36% and 49.53% respectively. 12-month prevalence of neck pain was found to be 31.27%, no more than the previous reported global prevalence of 37.2% [84]. In 2015, an EU-based study compiling survey data from workers about MSDs in the past 12 months and found that pain in the lower limbs was reported by 29%, upper limbs and neck by 41% and ‘backache’ by 43% [85]; thus farmers in LMIC are experiencing more lower limb symptoms, which may be explained by less reliance on mechanisation and longer spent in crouching postures or standing on uneven ground, known risk factors for the development of hip and knee pain [4].

A key strength of our study arises from the up-to-date comprehensive search strategy, which enables us to investigate the prevalence of pain across different MSK disorders in LMICs among people with different farming practices (such as pesticide use, organic farming, food, and flower growing and harvesting, tractor driving and subsistence farming). In addition, we followed the regress methodology to provide reliable estimates and critically appraised the quality of included studies. However, several limitations are present. In our study, limited information was available to explore factors associated with pain therefore, further studies are required to study pain and its prognosis in people with musculoskeletal disorders. Furthermore, it was not possible to draw any conclusion on the association by farming type due to farmers in the included studies might have been engaged in multiple different farming activities, for example, farmers that maintained livestock also maintained various crops. We did not include fishing in our inclusion criteria as some rural population could have been engaged in both fishing and farming. It is therefore possible we may have missed some useful studies. The inclusion criteria also included study participants aged 18 years and over. Therefore, some younger farming population might have been missed from this systematic review. Whilst we did not set out to research only non-inflammatory or mechanical causes of musculoskeletal pain and we used a broad search strategy to include musculoskeletal disorders from all aetiologies, the evidence base for agricultural workers appears to be exclusively relating to non-inflammatory causes of musculoskeletal pain.

As we approach the United Nation’s 2030 Agenda for Sustainable Development [86] deadline and a goal of ending poverty and hunger everywhere, it is essential that the health and wellbeing of agricultural workers in LMIC is supported. There is a high prevalence of musculoskeletal disorders amongst this population, and we recommend low back pain in agricultural workers living in Africa is a priority area for future research given its relatively high 12-month prevalence compared to that seen in other LMIC regions. The reasons for this are likely to be multifactorial and include both mechanical and psychosocial factors. Given the importance of agriculture in LMICs, its contribution to employment, Gross Domestic Product, and food security, it is important that employers and healthcare providers work together to encourage the earlier identification of problems, especially those that may become persistent, and to develop and implement cost-effective care pathways to support individuals, prevent disability and reduce loss of productivity.

### Supplementary Information

Below is the link to the electronic supplementary material.

Supplementary file1 (DOCX 189 KB)

## Data Availability

Data is available from the corresponding author at reasonable request.
