# Supplementary material for: Musculoskeletal disorders and pain in agricultural workers in Low- and Middle-Income Countries: a systematic review and meta-analysis
Source: Rheumatol Int. 2023 Nov 24;44(2):235–47. doi: 10.1007/s00296-023-05500-5 (PMC10796632; doi:10.1007/s00296-023-05500-5)
Supplement: Supplementary file 1 — Supplementary file1 (DOCX 189 KB) [file 296_2023_5500_MOESM1_ESM.docx]

Supplementary Information

Contents

1. Table 1: Search strategy
2. Table 2: Summary characteristics and raw data MSD prevalence estimates
3. Figure 1: Funnel plot depicting publication bias for studies reporting 12-month low back pain prevalence
4. Table 3: Associations of MSDs as reported in prevalence studies
5. Table 4: Associated burdens of MSDs as reported in prevalence studies
6. Table 5: Quality appraisal of selected studies

Supplementary Information 1: table 1. Search strategy

Search strategy used in Medline, Embase, PsychINFO. Terms for MSD, agricultural workers and, low and middle income countries were combined with ‘and’ command.

| 1·Musculoskeletal Disease |
| --- |
| Exploded Medical Subject Heading (MeSH) terms and ‘multiple places’ command (·mp) for keywords: ‘pain’, ‘back’, ‘knee’, ‘shoulder’, ‘neck’, ‘elbow’, hand$’, ‘hip’, ‘foot’, ‘feet’, ‘musculoskeletal disease’·    /mp command for ‘osteoarthr$’, ‘arthr$’, ‘arthral$’, ‘arthro?s’, ‘chronic adj2 pain’    Terms pertaining to pain and MSDs were combined using the ‘or’ command |
| 2· Agricultural workers |
| The ·mp command was used for terms: ‘farm’, ‘farms’, ‘farmer$’, ‘farming’, ‘farmhand$’, ‘farmworker$’, and ‘agricultur’·    Agriculture-related terms were combined with the ‘or’ command· |
| 3· Low and Middle Income Countries |
| Exploded Medical Subject Heading (MeSH) terms for ‘Developing Countries’, and using combinations of middle income / low income / poor income / less developed / least developed / developing / under developed or underdeveloped / 3^rd^ world / third world / LAMI / LMIC / countr* / nation / nations / econom*    LMIC regions and all countries and name-variants listed by the World Bank in 2018 were also included and combined with ‘or’ command: ‘Asia’, ‘Middle East’, ‘Far East’, ‘Africa’, ‘Sahara’, ‘Caribbean’, ‘West Indies’, ‘Latin America’, ‘Afghanistan’, ‘Benin’, ‘Burkina Faso’, ‘Burundi Central African republic’, ‘Chad’, ‘Congo’, ‘Eritrea’, ‘Ethiopia’, ‘Gambia’, ‘Guinea’, ‘Guinea-Bissau’, ‘Haiti’, ‘North Korea’, ‘Democratic People’s republic of Korea’, ‘Liberia’, ‘Madagascar’, ‘Malawi’, ‘Mozambique’, ‘Nepal’, ‘Bangladesh’, ‘Bhutan’, ‘Bolivia’, ‘Cabo Verde’, ‘Cape Verde’, ‘Cambodia’, ‘Congo’, ‘Cote d Ivoire’, ‘Djibouti’, ‘Egypt’, ‘El Salvador’, ‘Ghana’, ‘Guatemala’, ‘Georgia’, ‘Honduras’, ‘India’, ‘Indonesia’, ‘Jordan’, ‘Kenya’, ‘Kiribati’, ‘Kosovo’, ‘Kyrgyz’, ‘Kyrgyzstan’, ‘Lao’, ‘Laos’, ‘Lesotho’, ‘Mauritania’, ‘Micronesia’, ‘Moldova’, ‘Mongolia’, ‘Myanmar’, ‘Nicaragua’, ‘Nigeria’, ‘Pakistan’, ‘Papua new guinea’, ‘Philippines’, ‘Samoa’, ‘Sao tome’, ‘Principe’, ‘Solomon island’, ‘Sri Lanka’, ‘Sudan’, ‘Swaziland’, ‘Eswatini’, ‘Syria’, ‘Syrian Arab Republic’, ‘Tajikistan’, ‘Timor’, ‘Tunisia’, ‘Ukraine’, ‘Uzbekistan’, ‘Vanuatu’, ‘Vietnam’, ‘West Bank’, ‘Gaza’, ‘Yemen’, ‘Zambia’, ‘Albania’, ‘Algeria’, ‘American Samoa’, ‘Argentina’, ‘Azerbaijan’, ‘Belarus’, ‘Belize’, ‘Bosnia’, ‘Herzegovina’, ‘Botswana’, ‘Brazil’, ‘Bulgaria’, ‘China’, ‘Colombia’, ‘Costa Rica’, ‘Croatia’, ‘Cuba’, ‘Dominica’, ‘Dominican republic’, ‘Equatorial Guinea’, ‘Ecuador’, ‘Fiji’, ‘Gabon’, ‘Grenada’, ‘Guyana’, ‘Iran’, ‘Iraq’, ‘Jamaica’, ‘Kazakhstan’, ‘Lebanon’, ‘Libya’, ‘Macedonia’, ‘Malaysia’, ‘Maldives’, ‘Marshall islands’, ‘Mauritius’, ‘Mexico’, ‘Montenegro’, ‘Namibia’, ‘Nauru’, ‘Panama’· ‘Paraguay’, ‘Peru’, ‘Romania’, ‘Russia’, ‘Russian’, ‘Serbia’, ‘South Africa’, ‘St Lucia’, ‘St Vincent and the grenadines’, ‘Suriname’, ‘Thailand’, ‘Tonga’, ‘Turkey’, ‘Turkmenistan’, ‘Tuvalu’, ‘Venezuela’, ‘Niger’, ‘Rwanda’, ‘Senegal’, ‘Sierra Leone’, ‘Somalia’, ‘South Sudan’, ‘Tanzania’, ‘Togo’, ‘Uganda’, ‘Zimbabwe’, ‘Angola’, ‘Armenia’ |

Supplementary Information 2: table 2. Summary characteristics and raw data MSD prevalence estimates

| **Study Author, Country** | **a)Participants (n) b) % male c) Age range** | **MSD:Prevalence** |
| --- | --- | --- |
| **AFRICA** | | |
| Birabi 2012; Nigeria | a) 310 b) 42·6 c) 36·71 (8·98, 18-58) | 12m low back pain prevalence: n=208 (67·1%) |
| Diallo 2020, Togo | a)46; b)70·8; c)22-62y (SD not stated) | No pain duration defined· Muscle pain n=31 (64·6%) |
| Fabunmi 2005; Nigeria | a)500; b) 52·2 c) 48 | 12-month prevalence of low back pain 72·4% (n=362) (203 (73·5%) males and 159 (71·0%) females) |
| McNeill; 1998; Ghana | a)100; b)100; c)not stated | No pain duration defined for n=100: low back pain 76%. An opportunity sample of n=40 reported point prevalence of lower back pain of 48% and 77% claimed they had suffered low back pain in the last 12 months |
| Mushayi; 2014; Zimbabwe | a)30; b) 70; c) 18-60y | 12-month prevalence: 23 (76·7%) reported musculoskeletal pain or discomfort in at least one of the nine anatomical body areas.  LBP n=15 (50%), upper limb n=10 (33·3%), shoulder n=5 (16·7%), wrist/hands n=4 (13·3%), lower limb n=7 (23·3%), ankles/foot n=3 (10%), hips/thighs n=2 (6·7%), knees n=2 (6·7%), neck n= 6 (20%). |
| Naidoo 2009; South Africa | a)911; b)0; c)No range available· Irrigation scheme mean age 40·6 (39·3-41·9 95% CI); drylands mean age 42·8 (41·7-43·8) | 12-month pain prevalence: 63·9-72·3%; upper extremities 668 (73·3%), lower extremities 613 (6·3%), back 582 (63·9%).  Prevalence of pain lasting longer than 3 months: 42·8-48·3%; upper extremities 440 (48·3%), lower extremities 415 (45·6%), back 390 (42·8%). |
| Omokhodion 2002; Nigeria | a)190 farmers (900 total population); b)not stated;c)20-85y | 12 month pain prevalence: low back pain 46% (compared to 40% of the total population). |
| Omokhodion 2004; Nigeria | a)27 farmers (474 total population) | 12 month pain prevalence: LBP 85% (n=23). Odds of LBP compared to the rest of the population is OR 4·06 (1·24-12·95, p=0·018) when adjusted for sex, educational status, smoking status and past history of trauma· |
| Tella 2013; Nigeria | a)604; b) 60·9; c) modal average 45-55y (range <25y to >75y) | 12-month prevalence of LBP: 84% (n=447); |
| Worku 2000; South Africa | a)4001; b)0; c) no information | Prevalence of LBP (no duration defined):  mild, moderate or severe LBP 58·49% (n=2340); severe: 10·12% (n=405); moderate: 12·82% (n=513); mild 35·54% (n=1422)· |
| **ASIA** | | |
| Barerro 2006; China | a)11,879; b) 49·0;  c)25-64 | 12m low back pain prevalence:  n= 7613 (66·9%).  Farmers had greater odds of LBP + other pain sites than other occupational groups in adjusted analyses (adjusted for age, group, gender BMI, occupation, smoking, education, time pressure, physical stress, vibration at work)   (farmers is reference group):  LBP alone: Administration/services: OR 0·5 (0·4-0·6), p<0·05; Manufacturer worker: 0·5 (0·4-0·7), p<0·05; Household: 0·6 (0·4-0·8), p<0·05; Other (including retired): 0·5 (0·4-0·7), p<0·05·  LBP + 1 other pain: Administration/services: 0·4 (0·3-0·6), p<0·05; Manufacturer worker: 0·5 (0·4-0·8), p<0·05; Household: 0·5 (0·4-0·7), p<0·05; Other (including retired): 0·6 (0·5-0·9), p<0·05  LBP + 2 other pains: Administration/services: 0·4 (0·3-0·6), p<0·05; Manufacturer worker: 0·4 (0·3-0·8), p<0·05; Household: 0·8 (0·5-1·1), NS  Other (including retired): 0·9 (0·9-1·3), NS·  LBP + 3 other pains: Administration/services: 0·3 (0·1-0·5), p<0·05; Manufacturer worker: 0·1 (0·0-0·5), p<0·05; Household: 0·5 (0·3-1·0), p<0·05; Other (including retired):1·0 (0·6-1·6),NS |
| Basher 2015; Bangladesh | a)200; b) 100; c) 20-60 | Joint pain and stiffness in the last 12 months: Any 65·5%; Knee pain 48·1%; Back pain 22·9%; Waist pain (low back ache) 13·3%; Neck pain 18·3%; shoulder pain 10·7%; Pain in different parts of the body at least once during work: 42% |
| Bhandari 2018; Nepal | a) 183; b) 89·6 c) <20 - >=60 | 19·1% reported ‘muscle pain’ after pesticide application |
| Bihari 2011; India | a) 2086 subjects total; no (n) for farmers; 51·6 (of n=2086); c) 10-70y (of n=2086) | Pain at interview time or during the preceding 24 hours: 31·4% male; 44·7% female; no associated CIs or p-values· |
| Chokprasit 2022; Thailand | a)317; b)51·1; c)28-60y (SD 7·20) | point-prevalence of LBP total: n=226 (71·29%); male: n=113 (35·6%); female: n=113 (35·6%) |
| Das 2013; India | a)85 farmers (and 85 non-farmers); b) % male not stated; c) 31·9 (+- 6·82) | No information on pain duration· Unadjusted OR of pain in farmers v controls:  Neck: farmers 48 (56·6%), controls 10 (11·8%), OR 9·72 (4·42-21·37), p<0·001; Shoulder: farmers 52 (61·2%), controls 8 (9·5%), OR 15·16 (6·49-35·44), p<0·001; Elbows farmers 18 (21·2%), controls 2 (2·4%), OR11·14 (2·49-49·76), p<0·001; Wrists farmers 20 (23·5%), controls 6 (7·0%), OR 4·05 (1·53-10·68), p<0·01; Hands: farmers 51 (60·0%), controls 9 (10·5%), OR 12·66 (5·60-28·64), p<0·001; Upper back: farmers 40 (47·1%), controls 10 (11·8%), OR 6·66 (3·03-14·62), p <0·001; Lower back: farmers 84 (98·8%), controls 30 (35·3%), OR 154·00 (20·4-1162·30), p<0·001; Knees: farmers 78 (91·8%), controls 28 (32·9%), OR 22·68 (9·26-55·56), p<0·001; Ankles: farmers 56 (65·9%), controls 16 (18·8%), OR 8·32 (4·11-16·85), p<0·001; Feet: farmers 22 (25·9%), controls 18 (21·2%); NS |
| Das 2015; India | a)220; b)50 c) Men: 35·2 (7·12) Women: 34·3 (4·66) | Pain, numbness, stiffness, swelling and tingling in different body parts during work, after work, at night or for longer than 24 hours:  Males:90% reported pain; Females: 98% reported pain·  prevalence of tingling, numbness, stiffness and swelling (‘discomfort’): Neck n=116 (52·7%); shoulder n=81 (60·9%); elbow: 73 (33·2%) ; wrists: 88 (40%); hands: 118(53·6%); upper back: 51 (23·2%); lower back: 197 (89·5%); knees: 178 (81·0%); ankles: 60 (27·3%); feet: 64 (29·1%) |
| Dianat 2020, Iran | a) 377 farmers; b) Men: n=149 (39·5%); c)18-69 years (SD 11·1) | 12-month prevalence of pain in any of 9 body sites: Males n= 123 (82·6%), females n= 201 (88·2%), All n=324 (85·9%).  12-month prevalence of pain (All farmers): neck n=226 (59·9%), shoulders n=165 (43·8%), elbows n=68 (18%), hands/wrist n=136 (36·1%), upper back n=232 (61·5%), low back n=283 (75·1%) hips/thighs/buttocks n=150 (39·8%), knees n=234 (62·1%), ankles/feet n=103 (27·3%), any region n=324 (85·9%)·  Male farmers: neck n=78 (64·9%), shoulders n=55 (36·9%), elbows n=16 (10·7%), hands/wrist n=46 (30·9%), upper back n=84 (56·4%), low back n=103 (69·1%) hips/thighs/buttocks n=43 (28·9%), knees n=86 (57·7%), ankles/feet n=34 (22·8%).  Female farmers: neck n=148 (52·3%), shoulders n=110 (48·2%), elbows n=52 (22·8%), hands/wrist n=90 (39·5%), upper back n=148 (64·9%), low back n=180 (78·9%) hips/thighs/buttocks n=107 (46·9%), knees n=148 (64·9%), ankles/feet n=69 (30·3%) |
| Emami 1998; Iran | a)200 farmers (1000 total, from 5 different occupational groups) b) 0 c) 33·5y (25-45) of n=1000 | one year prevalence in farmers 21% (workers 17%, housewives 12%, nurses 9·5%, clerks 4%); (difference between groups p=0·0001)  lifetime prevalence in farmers was 35% (32·5% in manual workers, 28% housewives, 26% nurses, 15% in clerks); (difference between groups p=0·0001).  Current prevalence: farmers 10·5% (workers 10·5%, housewives 7%, nurses 2·5%, clerks 1%); (difference between groups p=0·0001) |
| Gupta 2013; India | a)301; b) % male not stated; c) 42·44 (SD 11·36) | Weekly and yearly prevalence combined: Neck: 10% (n=31); Shoulder: 22% (n=66); Lower back: 60% (n=180)· Knee: 39% (n=117); Elbow ca· 4% ; Wrist ca· 12%; Upper back ca·4%; Hip ca·4%; (estimates taken from bar chart)· No overall prevalence reported· |
| Hasan 2022, Bangladesh | a) 200; b) Male n=167 (83·50%); c) range 19-80 (SD 14·891) | Prevalence period not stated· Arthritis n=12 (6%) |
| Hongsibsong 2017; Thailand | a) n=150 including 103 farmers (Rice n=21; Corn n=33; Double-crop n=49); b) Rice 95·2; corn 87·); double crop 89·8; c) 52 - 55 (Rice 55 SD8 Corn 52 SD8 Double crop 52 SD7) | Point prevalence chest pain: farmers 5/103 (4·9%); non farmers 6/47 (12·8%): no significant difference between groups (OR 0·35 (0·101-1·207).  Point prevalence muscle weakness: farmers 32 (31·1%) and non-farmers 5 (10·6): significant difference OR 3·79 (1·370-10·466, p<0·01) |
| Jain 2018; India | a) 140; b) 81·4 c) males was 34·3y (SD 9·7); mean age females 38·4 (SR 10·4) | ‘trouble in various upper extremity body parts’ during the last 6 months: neck 59 (42·1%), shoulders 80 (57·1%), elbows/ forearms 66 (47·1%), wrists/hands 78 (55·7%), upper back 52 (37·1%), lower back 103 (73·6%), fingers 90 (64·2%), any site 109 (77·9%) |
| Kaewdok 2020, Thailand | a) 481; b) male n=197 (41%); c)>60 y (SD 7·1) | 7-day pain prevalence: n =423 (87·9%) and 12-month pain prevalence: n= 428 (88·9%)  7-day pain prevalence: lower extremity n= 307 (63·83%), low back n= 210 (43·66%), shoulders n= 148 (30·77%), upper back n= 114 (23·70%), neck n=94 (19·54%), arms n= 85 (17·67%), hands/wrists n=66 (13·72%), feet/ankles n=59 (12·27%).  12-month pain prevalence: lower extremity n=315 (65·49%), low back n=205 (42·62%), shoulders n=144 (29·94%), upper back n=115 (23·91%), neck n=89 (18·50%), arms n=81 (16·84%), hands/wrists n=60 (12·47%), feet/ankles n=53 (11·02%) |
| Kairi 2022, Bangladesh | a) 346; b) male n=70 (20·2%); c) 18-60y (SD not available) | 12-month MSS prevalence: n=280 (80·9%), 4 weeks MSS prevalence: n=277 (80·1%), point prevalence MSS: n=265 (76·6%)  12-month MSS prevalence in 9 body sites: neck: n= 90 (32·1%), shoulder n= 219 (78·2%), elbow n= 58 (20·7%), wrist/hand n= 84 (30%), upper, back n=157 (56·1%), lower back n=91 (32·5%), hip/buttock n=72 (25·7%), knee n=30 (10·7%), ankle n= 19 (6·8%), any body region n=280 (80·9%) |
| Kar 2007; India | a)400; b) 50; c) 19-65 | ‘Problem during work’: lower back 'problem' 48·8%; wrist-hand 'problems' 46·6%; pain in lower extremities 28·8%, shoulder (ca· 20% - no raw data 15provided to calculate, figure taken from bar chart), neck (ca·15% - no raw data provided to calculate, figure taken from bar chart) |
| Karimi 2020, Iran | a)48; b) male n=48; c) 18-47 (SD 7·5) | Pre-intervention pain prevalence: n=47 (97·9%). Post-intervention pain prevalence: n=41 (85·4%)·  Pre-intervention pain prevalence by site: neck n=18 (37·5%), shoulder right n=31 (64·6%), shoulder left n=22 (45·9%), upper back n=13 (27·1%), upper arm right n=1 (2·1%), upper arm left n=1 (2·1%), lower back n=33 (68·8%), forearm right n=12 (25·0%), forearm left n=12 (25·0%), wrist right n=6 (12·5%), wrist left n=6 (12·5%), hip/buttocks n=3 (6·3%), thigh right n=7 (14·6%), thigh left n=7 (14·6%), knee right n=13 (27·1%), knee left n=13 (27·1%), lower leg right n=11 (22·9%), lower leg left n=11 (22·9%), foot right n=0 (0%) and foot left n=0 (0%)·  Post-intervention pain prevalence by site: neck n=12 (25·0%), shoulder right n=17 (35·5%), shoulder left n=16 (33·3%), upper back n=9 (18·5%), upper arm right n=0 (0%), upper arm left n=0 (0%), lower back n=20 (41·7%), forearm right n=5 (10·4%), forearm left n=4 (8·3%), wrist right n=2 (4·1%), wrist left n=2 (4·1%), hip/buttocks n=2 (4·1%), thigh right n=4 (8·3%), thigh left n=4 (8·3%), knee right n=6 (12·5%), knee left n=7 (14·6%), lower leg right n=9 (18·5%), lower leg left n=9 (18·5%), foot right n=0 (0%) and foot left n=0 (0%)· |
| Kaur 2022, India | a) 200; b) male n=0; c) 20-50y (SD 8·10) | 12-month prevalence MSK discomfort: Neck 18 (9·0%), Shoulder 33 (16·5%), elbow 13 (6·5%), hand 19 (9·5%), upper back 4 (2·0%), lower back 114 (57·0%), thighs/hip 5 (2·5%), knee 61 (30·5%), ankle/foot 5 (2·5%)· 7-day prevalence: Neck 18 (9·0%), Shoulder 33 (16·5%), elbow 13 (6·5%), hand 19 (9·5%), upper back 4 (2·0%), lower back 114 (57·0%), thighs/hip 6 (3·0%), knee 60 (30·0%), ankle/foot 5 (2·5%) |
| Keawduangdee 2015; Thailand | a)344; b) and c) no information | Low back pain in the last 24 hours: 83·14% (95% CI 79·16-87·11) |
| Kongtawelert 2022, Thailand | a)603; b) male n=250 (41·5%); c) 18-79 (SD 11·6) | Planting season pain prevalence(n=603): Head n=22 (3·6%), neck n=29 (4·8%), shoulder n=61 (10·1%), elbow n=22 (3·6%), wrist n=64 (10·6%), upper back n=35 (5·8%), lower back n=97 (16·1%), hip n=41 (6·8%), knee n=118 (19·6%), foot n=25 (4·1%)·  Harvesting season pain prevalence (n=603): head n=10 (1·7%), neck n=28 (4·6%), shoulder n=138 (22·9%), elbow n=44 (7·3%), wrist n=120 (19·9%), upper back n=44 (7·3%), lower back n=224 (37·1%), hip n=50 (8·3%), knee n=173 (28·7%), foot n=29 (4·8%)· |
| Kumar 1999; India | a)100; b) not reported; c)25-45 | ‘Back17 problems’ in the last 2 weeks, last 12 months and lifetime· Any reported back symptom n=44 (44%)·  28 (56%) of tractor driving farmers (TDF) ha18d back symptoms, compared with 16 (32%) of non-tractor driving farmers (NTDF) (p=0·015)·   'Regular back ache' 20 TDF (40%) and 9 NTDF (18%19) p=0·015·  Work-related backache more prominent in TDFs 21 (42%) v 11 (22%) TDFs· |
| Liu 2012; China | a)2045; b)52·5; c)15-84 | 786 farmers (2038·4%) reported general back pain in the previous 3 months; 518 (25·4%) reported back pain that affected work· |
| Manothum; 2018; Thailand | a)320; b) 80·3%; c) 52·4 SD9·8 | No pain duration defined· No(%) Mild(%) Moderate(%) Severe(%):·Neck 104(32·5) 101(31·6) 97(30·3) 18(5·6); Shoulder 55(17·2) 177(55·3) 83(25·9) 5(1·6); Upper back 29(9·1) 121(3·8) 166(51·9) 4(1·3); Lower back 27(8·4) 102(31·9) 50(15·6) 141(44·1); Upper arm 65(20·3) 107(33·4) 139(43·4) 9(2·8); Elbow 103(32·2) 105(32·8) 104(32·5) 8(2·5); Lower arm 63(19·7) 153(47·8) 67(20·9) 37(11·6); Hand 26(8·1) 82(25·6) 87(27·2) 125(39·1); Thigh 67(20·9) 150(46·9) 86(26·9) 17(5·3); Knee 25(7·8) 75(23·4) 118(36·9) 102(31·9); Calf 29(9·1) 107(33·4) 156(48·8) 28(8·8); 1Foot 16(5·0) 72(22·5) 178(55·6) 54(16·9) |
| Meksawi; 2012; Thailand | a)427; b)66·7; c)38 SD 9·2 | Pain in the previous 3 months: upper arms 8·9% (6·4-12·0); lower arms 2·1% (1·0-4·0); wrists 2·3% (1·1-4·3); neck 3·0% (1·6-5·2); back 52·9% (48·1-57·7); legs (14·8% (11·5-18·5) |
| Momeni 2020, Iran | a) 1501; b) not available c) 16-70 (SD 13·64) | MSK symptoms during last 12 months (n=1501): Neck n=548 (36·5%), shoulders n=545 (36·2%), elbows n=330 (21·98%), wrists/hands n=522 (34·77%), upper back n=550 (36·6%), lower back n=890 (59·3%), thighs n=223 (14·9%), knees n=554 (36·9%), ankles/feet n=349 (23·3%) |
| Naeini 2020; Iran | a)19; b) not stated;c )not stated | 12-month prevalence: Neck 5%; ankles 5%; Knee 21%; Foot 0%; Low back 36%; Wrist 26%; Elbow 0%; Shoulder 15% |
| Nawi, 2016; Malaysia | a)88; b)100; c) modal age 28y (18-50, SD 6·5) | 12 month pain prevalence due to daily working activities: any body part: 100%; Always in pain: 11 (12%); Frequently in pain: 13 (15%); Sometimes in pain: 64 (73%).  LBP 99%; upper back 85%; shoulder 77%; buttock 81%; neck 74%; calf 71%; arm 58%; thigh 52%; wrist 46%; finger 40%; ankle 38%; foot 34%· |
| Ng 2014; Malaysia | a)143; b) not stated; c)28·30y (SD 7·24) | 12 month pain prevalence: any body part 93·0%; neck 32·2%, shoulder 32·2%, upper back 28·0%, lower back 58·0%, elbow 20·3%, hand/arms 26·6%, thigh 21·0%, knee 45·5%, ankle / feet 25·2%·  7-day pain prevalence: any body part 43·4%; neck 11·2%, shoulder 9·8%, upper back 9·8%, lower back 24·5%, elbow 6·3%, hand/arms 6·3%, thigh 5·6%, knee 14·0%, ankle/feet 4·9%· |
| Ng 2015; Malaysia | a)446; b) 100; c) 24·48 (SD 6·893) | 7-day pain prevalence: Any body part 45%; Neck 13%; Shoulder 13%; Upper back 8%; Elbow 7%; Lower back 28%; Hand/arm 6%; Thigh 6%; Knee 15%; Foot 6%·  12 months: Any body part: 86%; Neck 26%; Shoulder 28%; Upper back 21%; Elbow 18%; Lower back 58%; hand/arm 19%; thigh 16%; knee 40%; foot 19%· |
| Phajan 2014; North-Eastern Thailand | a)540; b)42·41%; c)44·75 (SD 7·67) | 7 day pain prevalence: any pain 448 (82·96%), 1 site 25·67%, 2 sites 30·80%, 3 sites 18·08%, 4 sites 11·16%, 5 sites 5·58%, 6 sites 6·03%, 7 sites 1·56%, 8 sites 0·89, 9 sites 0·22%; 12 months: any pain 479 (88·70%), 1 site 24·84$, 2 sites 26·72%, 3 sites 17·33%, 4 sites 11·27&, 5 sites 8·77%, 6 sites 4·80%, 7 sites 4·38%, 8 sites 1·25%, 9 sites 0·63%· |
| Reddy 2012, India | a)343; b) not stated; c) 48·04y (28-62y, SD 6·51) | Pain in the last 12 months lasting 3 months or more: Neck pain 72·2%, low back pain 66·2%, shoulder pain 44·9%, knee pain 55·8%, ankle / foot pain 34·4%, elbow pain 33·2%, upper back pain 30·8%, wrist pain 50·1%, hip/thigh pain 15·3%· |
| Meucci; 2015; Brazil | a)2468; b)59·3; c)18-50y older | chronic low back pain prevalence 8·4% (n=207).  Pain in the past 1 month = 36·0% (n=888), acute back pain = 28·2% (n=696) |
| Milani; 2012; Brazil | a)204; b) not stated; c)32·3y | 12 month prevalence: 41% reported pain in at least one body part; neck 14·2% (n=29), shoulders 8·3% (n=17), Upper back 15·7% (n=32), elbows 1·0% (n=2), wrists/hands 6·4% (n=13), lower back 13·7% (28), hips/thighs 4·9% (n=8), knees 14·2% (n=29), ankles/feet 10·3% (n=21)  7 day prevalence: neck 1·5%, shoulder 1·5%, upper back 3·0%, elbows 0·0%, wrists/hands 2·9%, lower back 3·9%, hip/thighs 2·0%, knees 4·4%, ankles/feet 1·5% |
| Moreira; 2015; Brazil | a)22,410 agricultural workers & 804 non-agricultural workers; b)73·2 (n=23,214); c) 18-60y (n=23,214) | No pain duration defined·  Odds ratios of conditions in agricultural workers compared to baseline of non-agricultural workers· Prevalence of spine or back pain 21·1% (compared to 15·8% in non-agricultural workers, Odds ratio 1·42 (1·15-1·75))· Prevalence of arthritis or rheumatism 7·7% (compared to 4·5% in nonagricultural workers, odds ratio 1·79 (1·27-2·54)· Prevalence of tendonitis and tenosynovitis 1·5% (compared with 2·3% for non-agricultural workers, odds ratio 0·63 (0·37-1·08)· |
| Razavi, 2014; Iran | a)350; b) 98; c) 22-88y | Knee: 12 month prevalence: 58% / 1 month: 50% / 7d: 58%;  Hand & wrist: 12m: 33% / 1m: 31% / 7d: 30%; Lower back: 12m: 31% / 1m: 21% / 7d: 20%; Foot & ankle: 12m: 31% / 1m: 29% / 7d: 31%; Shoulder: 12m 28%/ 1m: 20% / 7d: 19%; upper back: 12m: 24% / 1m: 17% / 7d: 17%; Neck: 12m: 23% / 1m: 13% / 7d: 11%; Hip and thigh: 12m: 21% / 1m: 23% / 7d: 21%; Elbow: 12m:19% / 1m: 13% / 7d: 13%· |
| Sahu, 2013; India | a)124; b)100; c)18-45y | Pain prevalence at the end of a day of work: 72% (n=89) pain in any body part· |
| Shan 2012; Malaysia | a)419; b)100; c)53·01 (SD 4·90) | 12-month pain prevalence: 12 month pain prevalences: Neck 59·9%, LBP 56·3%, shoulder 54·9%, knee 45·8%, ankles/feet 34·4%, elbow 33·2%, upper back 30·8%, wrist 30·1%, hips/thigh 5·3%· |
| Singh 2022, India | a)152; b)60·5%; c) 18-76y | MSK symptoms in one or more parts in 12 months: n=121 (79·6%)  MSK pain 12 month prevalence: Neck n=71 (46·71%), Shoulder n=70 (46·05%), elbows n=23 (15·13%), Wrist/hand n=42 (27·63%), upper back n=17 (11·18%), low back n=116 (76·32%), hips/thighs n=12 (7·89%), knees n=51 (33·55%), ankles/feet n=23 (15·13%). |
| Taechasubamorn 2011; Thailand | a)283; b)48·8; c)51·1 (29-72y) | Lifetime prevalence of LBP: 77·4%; 12-month prevalence of LBP: 56·2%; point prevalence of LBP: 49·1%· |
| Thetkathuek 2017; Thailand | a)861; b) 58; c) men 30·7y (SD 8·4); women 29·8 (SD 8·7) | At least one pain site: 692 (80·40%)·  LBP: 38·9% men, 44·7% of women, total 41·3%; upper back: 28·3% men, 28·1% women, total 28·2%; neck: men 23·8%, women 24·2%, total 23·9%; shoulder: 19·6% men, 24·4% women, total 21·6%; elbow: men 4·2%, women 4·7%, total 4·4%; wrist and hand: men 9·8%, women 14·4%, total 11·7%; hip and thigh: men 13·4%, 22·2% women, total 17·1%; knee 11·6% men, 16·1% women, 13·5% total; ankle 10·4% men, 12·2% women, 11·1% total· |
| Udom 2016; Thailand | a)433; b)32·2; c)45·14 (SD 10·68) | 12 month prevalence of LBP: 55·7% (n=241)·  24 hour pain prevalence: 33% (n=143)· |
| Vasanth 2015; India | a)195; b) ‘females large majority’; c) 45·6 (18-60y, SD 7·56) | 12-month pain prevalence: any pain 83·6%; Neck 49·1%; Shoulder 59·0%; Elbow 41·5%; Wrist 25·9%; Upper back 37·3%; Lower back 58·0%; Hip 3·3%; Knee 43·9%; Ankle 7·1%.  7-day pain prevalence: any pain 78·5%; Neck 42·0%; Shoulder 45·8%; Elbow 24·5%; Wrist 23·6%; Upper back 26·8%; Lower back 52·8%; Hip 1·9%; Knee 38·2%; Ankle 6·6%· |
| Xie 2020; China | a)1368 (392 ‘high exposure’ and 976 ‘low exposure’ giving 392 matched pairs); b) not stated; c) not stated· | pain and swelling of fingers or toes of unknown origin (raw data before matching), no further definition provided: Nothing (low exposure: 774; high: 314), light (low: 157, high: 56), middle (low: 44, high: 22)·  Pain, swelling and weakness in the joints of hands or feet: nothing (low: 713, high: 267); light (low: 180, high 86); middle (low: 83, high:38); muscle soreness and pain in the whole body: nothing (low: 669, high: 266), light (low: 218, high:88), middle (low:89; high: 38)· |
| Zhou 2021, China | a)182 valid answers; b)47·8%; c)31-60y (no SD provided) | Musculoskeletal symptoms: n=53 (29·1%). Duration of symptoms not specified· |
| **CENTRAL AND SOUTH AMERICA** | | |
| Barerro 2012; Columbia | a) 158; b) 27·5; c) 35·7 (20-60; 8·9) | Point prevalence: Carpal tunnel syndrome 32·9%; Medial or lateral epicondylitis 15·2%; De Quervain’s 13·3% |
| Caminiti Tejada 2011; Peru | a)268; b)85; c)47·27 (SD 13·82) | Point prevalence: lumbar pain 8%; joint pain 5%· |
| Carvalho 2020, CruzeiroSzortyka 2021, Fassa 2020; Brazil | a)2469; b) 59%; c)18-60y older (no SD given) | Carvalho 2020· 12-month prevalence of thoracic spine pain in females: n=212 (21·1%); males: n=312 (21·3%)  CruzeiroSzortyka 2021: 12-month prevalence of low back pain n=207 (8·3%)  Fassa 2020: 12-month prevalence of neck pain in females n=91 (9·1%), males n=91 (6·2%) |
| PinargoteCedeno 2021, Ecuador | a)106 farmers; b)91·51% c)18y and older (no SD given) | Farmers 12 month prevalence of pain: Neck n =20 (18·87%), shoulder n=58 (54·72%), thoracic spine n=14 (13·21%), lumbar spine n=45 (42·45%)  Farmers 7 day prevalence of pain: Neck n=14 (13·21%), shoulder n=36 (33·96%), thoracic spine n=6 (5·66%), lumbar spine n=25 (23·58%) |
| Rocha 2014; Brazil | a)259; b)57·1; c)51·2y (no range or SD given) | Pain during completion of work indicated on diagram, no further definition. 85·3% (n=221) complained of pain· |
| Rojas, 2105; Costa Rica, El Salvador, Guatamala, Honduras, Nicaragua, Panama | a)3544 agricultural workers (study population 12,024); b) 51·7-63·3% across the six countries· c) 18y and older | Cervical-dorsal: Costa Rica: 21·2 (17·1-25·4)%; El Salvador 55·5 (49·9-60·6)%; Guatamala 38·9 (35·7-42·2)%; Honduras 40·7 (37·3-44·1)%; Nicaragua 45·6 (42·1-49·2)%; Panama 17·9 (13·4-22·2)%·  Lumbar: Costa Rica: 30·7 (26·2-35·3)%; El Salvador 35·9 (31·2-40·8)%; Guatamala 16·4 (13·9-18·9)%; Honduras 25·3 (22·3-28·2)%; Nicaragua 31·1 (27·7-34·4)%; Panama 17·3 (12·8-21·7)%·  Upper limb pain: Costa Rica: 16·5 (12·9-20·1)%; El Salvador 51·7 (46·3-57·1)%; Guatamala 29·9 (26·9-33·1); Honduras 43·6 (40·2-47·1)%; Nicaragua 44·8 (41·2-48·4)%; Panama 18·1 (13·5-22·6)%·  Total any pain: Costa Rica: 44·4 (39·9-48·9)%; El Salvador 70·8 (65·7-76·0)%; Guatamala 56·7 (53·4-60·0)%; Honduras 63·1 (59·8-66·5)%; Nicaragua 68·2 (64·8-71·6)%; Panama 42·3 (35·3-47·1)% |
| Silva 2017; Brazil | a)185; b)45·4; c)44·24 (SD 10·83) | Undefined low back pain prevalence: 97·8 % (n=181) |
| Simas 2020, Brazil | a)36; b) 94·4%; c) ‘most were between 20-49 years’ | a)12-month pain prevalence, b) 7 day pain prevalence: Cervical (a) 33·3% (b) 30·8%; Shoulders (a) 47·2% (b) 2·9%; Elbows (a) 13·9% (b) 28·6%; Fists and/or Hands (a) 38·9% (b) 53·3%; Thoracic (a) 41·7% (b) 50·0%; Lumbar (a) 63·9% (b) 40·0%; Hips and/or Thighs (a)30·6% (b) 50·0%; Knees (a) 44·4% (b) 68·8%; Ankles and/or Feet (a) 27·8% (b) 60·0%· |

Supplementary Information 3: Figure 1: Funnel plot depicting publication bias for studies reporting 12-month low back pain prevalence


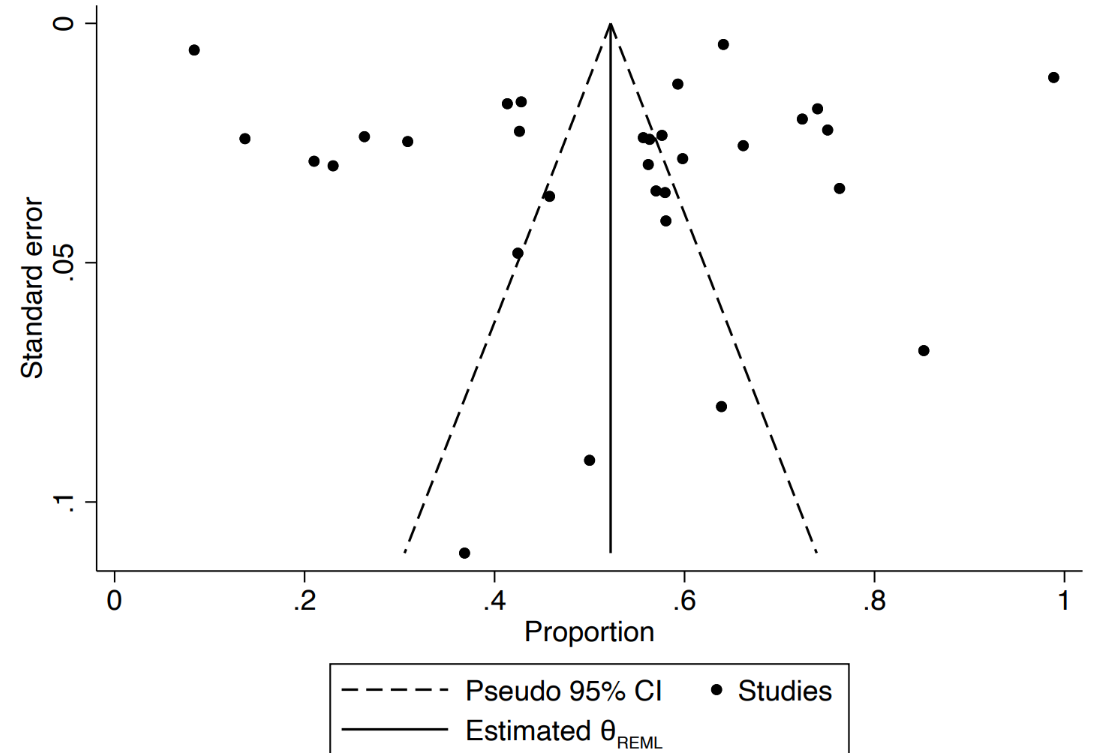


Supplementary Information 4: table 3· Associations of MSDs as reported in prevalence studies

| **Study Author, Country** | **Associations** |
| --- | --- |
| Basher 2015; Bangladesh | Most of the farmers who reported MSD were 41-60y old·  Those worked more than 20 years reported a higher prevalence of pain in any site (82·6%)  Those working 6 hours /day reported a higher prevalence of pain in any site (66·7%)  Most common position for reporting pain was squatting (52%)  Most common task to report pain in was weeding of plants (31%) |
| Birabi 2012; Nigeria | Aged 31-40 years (49·04%)  compared to ages <30 (15·38%), 41-50 (27·88%) and 51-60 (7·70%), p<0·001;  females (50·96%) compared to males (49·04%), p=0·001  Education: NS  Years of practice:  5-10y (20·19%), 11-15y (18·27%), 16-20y (19·23%), 21-25y (18·75%), 26-30y (11·06%), 31-35y (8·65%), >35y (3·85%), p<0·001;  Height (m): 1·46-1·50 (4·81%), 1·51-1·55 (31·25%), 1·56-1·60 (36·06%), 1·61-1·65 (16·83%), 1·66-1·70 (2·88%), 1·71-1·75 (5·77%), 1·71-1·80 (2·40%, p=0·02;  BMI: NS |
| Carvalho 2020, Brazil | Thoracic spine pain in previous year prevalence and associated factors in female tobacco farmers (n=1005)  smokers: 34·2%, aPR 1·71 (95% CI 1·22-2·39, p=0·002)  Cleaning equipment used to apply pesticides: 27·4%, aPR 1·40 (95% CI 1·09-1·81, p=0·009)  Pesticide poisoning (life): 43·1%, aPR 1·82 (95% CI 1·32-2·50, p<0·001)  Working hours during harvest 9-12 hours/day: 17·4%, aPR 0·62 (95% CI 0·48-0·81, p=0·002)  Prolonged work while sitting: 18·5%, aPR 0·75 (95% CI 0·59-0·95, p=0·019)  Green Tobacco sickness up to 3 episodes4 episodes in previous years: 35·5%, aPR 1·42 (95% CI 1·05-1·92, p=0·024)  Wheezing with dyspnoea (previous year): 34·1%, aPR 1·61 (95% CI 1·05-2·45, p=0·028)  Minor Psychiatric disorders: 38·5%, aPR 1·71 (95% CI 1·31-2·22, p<0·001)  Thoracic spine pain in previous year prevalence and associated factors in male tobacco farmers (n=1464)  Schooling 5-8 years: 24·7%, aPR 1·56 (95% CI 1·26-1·93) Schooling 9 years or more: 27·3%, aPR 1·76 (95% CI 1·18-2·62) p<0·001  Time working with tobacco 10-19 years· 20 years or more·  Lopping trees frequently/always  Lifting sticks  Cleaning equipment used to apply pesticides  Working hours during harvest  Working in a bending position  Green tobacco sickness  Wheezing  Minor Psychiatric disorders |
| Chokprasit 2022, Thailand | Multivariate analyses of risk factors associated with LBP of rubber farmers -  Working experience: AOR 1·519 (0·867-2·662, 95% CI, p=0·144)  Sufficient income: AOR 0·419 (0·220-0·799, 95% CI, p=0·008)  Stress/anxiety: AOR 1·256 (0·680-2·319, 95% CI, p=0·467)  Inheritance career: AOR 1·612 (0·885-2·936, 95% CI, p=0·119)  Agricultural registration: AOR 2·218 (1·132-4·346, 95% CI, p=0·020)  Work without training (habitual work): AOR 1·966 (0·990-3·904, 95%CI, p=0·054)  Squatting: AOR 1·329 (0·672-2·630, 95% CI, p=0·413)  Heavy workload (continue harvesting for ≥7h): AOR 1·739 (0·976-3·099, 95% CI, p=0·061)  Get muscle tension from the body directly: AOR 0·600 (0·303-1·190, 95% CI, p=0·144)  Prolonged standing (8h consecutively): AOR 2·948 (1·546-5·618, 95% CI, p=0·001) |
| Dianat 2020, Iran | Multivariate analysis of risk factors associated with neck symptoms -  Female: OR 2·09 (95% CI 1·04-4·22, p<0·05)  Years worked as an agricultural worker 5-15 years: OR 2·75 (95% CI 1·45-5·14, p<0·01)  Years worked as an agricultural worker >15 years: OR 4·09 (95% CI 1·95-8·56, p<0·001)  Job satisfaction moderate: OR 0·62 (95% CI 0·31-1·22, p>0·05 (p value not specified))  Job satisfaction high: OR 0·25 (95% CI 0·07-0·91, p<0·05)  RULA grand score ≥7: OR 3·29 (95% CI 1·38-7·89), p<0·01)  Multivariate analysis of risk factors associated with upper back symptoms -  Daily working hours >8: OR 3·36 (95% CI 1·38-8·20, p<0·01)  Working very fast: OR 2·93 (95% CI 1·07-7·83, p<0·05)  RULA B score ≥7: OR 3·17 (95% CI 1·31-7·67, p<0·05)  Multivariate analysis of risk factors associated with lower back symptoms -  Occupation vegetable farmers: OR 0·35 (95% CI 0·10-1·24, p>0·05 (p value not specified))  Occupation greenhouse farmer: OR 0·33 (95% CI 0·14-0·98, p<0·05)  RULA B score ≥7: OR 3·18 (95% CI 1·26-8·02, p<0·05)  RULA grand score≥7: OR 3·36 (95% CI 1·40-8·04, p<0·01)  Multivariate analysis of risk factors associated with knee symptoms -  Years worked as an agricultural worker 5-15 years: OR 1·28 (95% CI 0·65-2·51, p>0·05 (p value not specified))  Years worked as an agricultural worker ≥15 years: OR 2·77 (95% CI 1·13 -6·77, p<0·05)  RULA B score ≥7: OR 2·29 (95% CI 1·01-5·25, p<0·05)  RULA grand score ≥7: OR 2·61 (95% CI 1·01-6·82, p<0·05) |
| Fabunmi 2005; Nigeria | daily LBP experienced by 141 (39·0%), weekly by 87 (24·0%) , monthly by 59 (16·3%), three monthly by 44 (12·2%) and once in 4 months or more by 31 (8·5%)· Longest farming duration has highest LBP: 51-60 years in farming 100% LBP, lowest duration has lowest LBP 1-10 years 15·8%· check p-value |
| Fassa 2020, Brazil | Adjusted prevalence ratios for associated risk factors in female workers-  Age 40-59 years: OR 1·73 (95% CI 1·15-2·60, p=0·023)  Age 60 years or over: OR 0·92 (95% CI 0·30-2·89, p=0·023)  Smoker or ex-smoker: OR 2·00 (95% CI 1·17-3·41, p=0·011)  Bailing of tobacco leaves: OR 2·05 (95% CI 1·19-3·52, p=0·010)  Use of heavy chainsaws: OR 2·05 (95% CI 1·08-3·86, p=0·027)  Work at an intense or fast pace for 4-7 months: OR 1·54 (95% CI 0·97-2·42, p=0·033)  Work at an intense or fast pace for over 8 months: OR 1·77 (95% CI 0·93-3·38, p<0·033)  Work in an inclined position: OR 3·27 (95% CI 0·77-13·91, p=0·109)  Up to 3 episodes of Green tobacco sickness in the previous year: OR 1·41 (95% CI 0·70-2·83, p<0·005)  Over 4 episodes of Green tobacco sickness in the previous year: OR 2·15 (95% CI 1·25-3·70, p<0·005)  Adjusted prevalence ratios for associated risk factors in male workers -  Age 40-59 years: OR 1·59 (95% CI 1·02-2·47, p<0·001)  Age 60 years or over: OR 3·20 (95% CI 1·80-5·69, p<0·001)  Smoker or ex-smoker: OR 1·49 (95% CI 0·98-2·28, p=0·063)  Soil preparation for planting tobacco: OR 0·63 (95% CI 0·39-1·02, p=0·062)  Use of heavy chainsaws: OR 1·68 (95% CI 1·13-2·49, p<0·009)  Placing tobacco leaves in bars: OR 1·31 (95% CI 0·88-1·95, p=0·183)  Work at an intense or fast pace for 4-7 months: OR 1·49 (95% CI 0·93-2·37, p=0·085)  Work at an intense or fast pace for over 8 months: OR 1·56 (95% CI 0·83-2·94, p=0·085)  Work sitting on the floor: OR 0·66 (95% CI 0·44-0·99, p=0·047)  Strenuous work: OR 1·50 (95% CI 0·97-2·32, p=0·067)  Pesticide poisoning in the previous year: OR 3·85 (95% CI 1·05-14·14, p=0·042)  Up to 3 episodes of green tobacco sickness in the previous year: OR 2·07 (95% CI 1·08-3·95, p=0·005)  Over 4 episodes of green tobacco sickness in the previous year: OR 2·21 (95% CI 1·05-4·65, p=0·005) |
| Hongsibsong 2017; Thailand | Measures of pesticide use:  Mean level of DAP in farmers with symptoms compared with farmers reporting no symptoms:  Muscle weakness: NS; Chest pain: NS  Mean level of AChE activity in farmers with symptoms compared with farmers reporting no symptoms:  Chest pain: symptoms mean is 8,424 (8,073-10,413); No symptoms mean is 10,764 (9,535-11,700), p=0·039. Muscle weakness: NS  Measures of DNA damage:  Mean tail length in farmers with symptoms compared to farmers with no symptoms: Chest pain: symptoms 6·6 (6·5-7·3), no symptom 6·4 (5·9-6·6) p=0·031· Muscle weakness: NS  Mean tail moment:  Chest pain: symptom 3·3 (3·3-3·5), no symptom 3·2 (3·0-3·3) p=0·030; Muscle weakness: NS |
| Jain 2018; India | Multivariate logistic regression for pain in different body sites:  Age: p<0·05 for: Upper back OR 1·06 (1·00-1·13); Wrist/hands OR1·12 (1·04-1·21); Fingers OR 1·14 (1·05-1·24); Elbow OR 1·14 (1·06-1·23)  Gender (being male, female is ref): p<0·05 for: Elbow/forearm OR 0·28 (0·09-0·86);  Hand domination: NS;  Farming experience (yr): NS;  Daily working in farm (hrs): NS  Perceived work fatigue: Low (ref), Moderate: NS, High: elbow/forearms OR 3·05 (1·05-8·72)  RULA / A score: (<=5 is ref):  >=6: Wrist/hands OR 3·29 (1·23-8·77); Fingers 4·66 (1·63-13·34)  Elbow/forearms 3·1 (1·12-8·60)  RULA / B score: (<=7 is ref): Neck OR 0·45 (0·20-0·98) |
| Kaewdok 2020, Thailand | Using agricultural tools/equipment: MSD n=408 (89·47%), No MSD n=48 (10·53%)· Adjusted OR 4·04 (1·18-13·79, 95%CI) p=0·026·  Prolonged static postures: MSD n=337 (87·99%), No MSD n=46 (12·01%)· Adjusted OR 3·81 (1·05-13·82, 95%CI) p=0·042  Manual material handling >10kg: MSD n=279 (91·18%), No MSD n=27 (8·82%)· Adjusted OR 2·87 (1·22-6·82, 95%CI) p=0·016·  Females with MSD n=261 (91·9%), without MSD n=23 (8·10%), adjusted OR 2·52 (1·19-5·36) p=0·016 |
| Kairi 2022, Bangladesh | MSS and type of work: Plucking n=231 (83·4%), non-plucking n=49 (71%), p=0·019  Neck MSS and Sex: Male n=11 (15·7%), female n=79 (28·6%), p=0·028  Neck MSS and type of work: Pluckers n=80 (28·9%), non-pluckers n=10 (14·5%), p=0·015  Shoulder MSS and type of work: pluckers n=183 (66·1%), non-pluckers n=36 (52·2%), p=0·032  Wrist/hand MSS and type of work: pluckers n=74 (26·7%), non-pluckers n=10 (14·5%), p=0·034  Lower back MSS and work hours per day: ≤7 hours n=5 (14·3%), 8 hours n=81 (26·8%), ≥9 hours n=5 (55·6%), p=0·036  Elbow MSS and overtime: yes n=8 (30·8%), no n=50 (15·6%), p=0·047  Hip/buttock MSS and overtime: yes n=10 (38·5%), no n=62 (19·4%), p=0·021 |
| Kar 2007; India | Specific rice cultivation tasks and three most commonly reported pain sites (no CI or p value reported):  Reaping: Back pain 92%, upper leg pain 52%, pain in the wrist/hand 48%  Uprooting of seedlings: low back 72%, knee/ankle 54%, shoulder 52%  Transplantation: lower back  84·0%, knee/ankle 60%, wrist/hand 52%  Binding of straws: wrist /hand 56%, lower back 20%, head 8%  Threshing: Upper leg 72%, Wrist/hand 44%, Shoulder 32% |
| Karimi 2020, Iran | Severity of symptoms  Slightly uncomfortable  Pre-intervention: neck n=12 (27·0%), shoulder right n=19 (39·5%), shoulder left n=16 (33·3%), upper back n=10 (20·8%), upper arm right n=1 (2·1%), upper arm left n=1 (2·1%), lower back n=8 (16·7%), forearm right n=8 (16·7%), forearm left n=8 (16·7%), wrist right n=6 (12·5%), wrist left n=6 (12·5%), hip/buttocks n=2 (4·1%), thigh right unavailable, thigh left unavailable, knee right n=2 (4·1%), knee left n=2 (4·1%), lower leg right n=6 (12·5%), lower leg left n=6 (12·5%), foot right unavailable, foot left unavailable  Post-intervention: neck n=11 (22·9%), shoulder right n=15 (31·2%), shoulder left n=13 (27·0%), upper back n=9 (18·5%), upper arm right unavailable, upper arm left unavailable, lower back n=15 (31·2%), forearm right n=2 (4·1%), forearm left n=2 (4·1%), wrist right n=2 (4·1%), wrist left n=2 (4·1%), hip/buttocks n=2 (4·1%), thigh right n=2 (4·1%), thigh left n=2 (4·1%), knee right n=5 (10·4%), knee left n=4 (8·3%), lower leg right n=5 (10·4%), lower leg left n=5 (10·4%), foot right unavailable, foot left unavailable  Moderately uncomfortable  Pre-intervention: neck n=3 (6·3%), shoulder right n=9 (18·5%), shoulder left n=4 (8·3%), upper back n=3 (6·3%), upper arm right unavailable, upper arm left unavailable, lower back n=8 (16·7%), forearm right n=4 (8·3%), forearm left n=4 (8·3%), wrist right unavailable, wrist left unavailable, hip/buttocks n=1 (2·1%), thigh right n=6 (12·5%), thigh left n=6 (12·5%), knee right n=6 (12·5%), knee left n=6 (12·5%), lower leg right n=3 (6·3%), lower leg left n=3 (6·3%), foot right unavailable, foot left unavailable  Post-intervention: neck n=1 (2·1%), shoulder right n=2 (4·1%), shoulder left n=3 (6·3%), upper back unavailable, upper arm right unavailable, upper arm left unavailable, lower back n=3 (6·3%), forearm right n=2 (4·1%), forearm left n=3 (6·3%), wrist right unavailable, wrist left unavailable, hip/buttocks unavailable, thigh right n=2 (4·1%) , thigh left n=2 (4·1%), knee right n=1 (2·1%), knee left n=2 (4·1%), lower leg right n=3 (6·3%), lower leg left n=3 (6·3%), foot right unavailable, foot left unavailable  Very uncomfortable  Pre-intervention: neck n=2 (4·1%), shoulder right n=3 (6·3%), shoulder left n=2 (4·1%), upper back unavailable, upper arm right unavailable, upper arm left unavailable, lower back n=17 (35·4%), forearm right unavailable, forearm left unavailable, wrist right unavailable, wrist left unavailable, hip/buttocks unavailable, thigh right n=1 (2·1%), thigh left n=1 (2·1%), knee right n=5 (10·4%), knee left n=5 (10·4%), lower leg right n=2 (4·1%), lower leg left n=2 (4·1%), foot right unavailable, foot left unavailable  Post-intervention: neck unavailable, shoulder right unavailable, shoulder left unavailable, upper back unavailable, upper arm right unavailable, upper arm left unavailable, lower back n=2 (4·1%), forearm right unavailable, forearm left unavailable, wrist right unavailable, wrist left unavailable, hip/buttocks unavailable, thigh right unavailable, thigh left unavailable, knee right unavailable, knee left n=1 (2·1%), lower leg right n=1 (2·1%), lower leg left n=1 (2·1%), foot right unavailable, foot left unavailable |
| Keawduangdee 2015; Thailand | Prevalence of low back pain was higher in those reporting high or severe stress levels compared to those reporting mild or moderate levels of stress (aOR 2·2 (1·2-4·1, p<0·05, adjusted for number of working days, age and level of stress)  Prevalence of LBP was lower in those aged 45y or less working more than 5 days in a field (aOR 0·2 (0·1-0·6) |
| Kongtawelert 2022, Thailand | Univariate analysis of factors associated with any MSDs  Planting season:  Age 31-60: PR 2·36 (95% CI 1·31-4·24, p=0·004)  Age ≥61: PR 2·75 (95% CI 1·50-5·01, p=0·001)  Female: PR 1·25 (95% CI 1·05-1·48, p=0·011)  BMI <18·50: PR 1·08 (95% CI 0·70-1·68, p=0·732)  BMI ≥ 25·00: PR 1·06 (95% CI 0·90-1·25, p=0·507)  Years of work in tobacco farming (year): PR 1·02 (95% CI 1·01, 1·02, p<0·001)  Harvesting season:  Age 31-60: PR 1·38 (95% CI 0·98-1·94, p=0·062)  Age ≥61: PR 1·76 (95% CI 1·24-2·48, p=0·001)  Female: PR 1·16 (95% CI 1·02-1·31, p=0·024)  BMI <18·50: PR 1·21 (95% CI 0·91-1·60, p=0·193)  BMI ≥ 25·00: PR 1·11 (95% CI 0·99-1·26, p=0·080)  Years of work in tobacco farming (year): PR 1·01 (95% CI 1·01, 1·02, p<0·001)  Factors related to MSDs in the planting and harvesting seasons adjusted by age, gender and BMI·  Any MSD Planting season-  Seedling 4-6h: aPR 0·76 (95% CI 0·63-0·91, p=0·003)  Seedling 1-3h: aPR 0·84 (95% CI 0·66-1·07, p=0·156)  Plowing using hand tractor: aPR 1·08 (95% CI 0·74-1·58, p=0·682)  Digging holes manual: aPR 1·08 (95% CI 0·73—1·60, p=0·692 )  Planting 4-6h: aPR 1·00 (95% CI 0·82-1·23, p=0·973)  Planting 1-3h: aPR 0·89 (95% CI 0·69-1·15, p=0·372)  Crop maintenance spraying with backpack: aPR 0·77 (95% CI 0·64-0·92, p=0·005 )  Topping the tobacco plant 4-6h: aPR 0·74 (95% CI 0·62-0·89, p=0·001 )  Topping the tobacco plant 1-3h aPR 0·68(95% CI 0·50-0·91, p=0·010 )  Any MSD Harvesting season-  Carrying from the field (loading, group work): aPR 0·81 (95% CI 0·66-0·98, p=0·029)  Carrying from the field (unloading, group work): aPR 0·69 (95% CI 0·58-0·83, p<0·001)  Piercing/Threading group work: aPR 0·72 (95% CI 0·61-0·85, p<0·001 )  Curing in the burn (hanging up, group work): aPR 0·83 (95% CI 0·68-1·01, p=0·070)  Curing in the barn (climbing down, group work): aPR 0·73 (95% CI 0·61-0·88, p=0·001)  Bailing small size group work: aPR 0·83 (95% CI 0·71-0·96, p=0·014)  Bailing medium size group work: aPR 0·79(95% CI 0·68-0·93, p=0·003)  Bailing large size group work: aPR 0·55 (95% CI 0·47-0·65, p<0·001)  Transportation loading group work: aPR 0·67 (95% CI 0·56-0·79, p<0·001)  Transportation unloading group work: aPR 0·70 (95% CI 0·58-0·85, p<0·001)  Shoulder Planting season-  Seedling 4-6h: aPR 0·99 (95% CI 0·52-1·88, p=0·973)  Seedling 1-3h: aPR 1·93 (95% CI 0·96-3·89, p=0·066)  Plowing using hand tractor: aPR 1·80 (95% CI 0·79-4·08, p=0·161)  Digging holes manual: aPR 2·92 (95% CI 1·49-5·72, p=0·002)  Planting 4-6h: aPR 0·87 (95% CI 0·46-1·62, p=0·649)  Planting 1-3h: aPR 1·26 (95% CI 0·65-2·42, p=0·494)  Crop maintenance spraying with backpack: aPR 0·99 (95% CI 0·58-1·69, p=0·965)  Topping the tobacco plant 4-6h: aPR 1·06 (95% CI 0·58-1·94, p=0·852)  Topping the tobacco plant 1-3h: aPR 1·46 (95% CI 0·69-3·08, p=0·324)  Shoulder Harvesting season-  Carrying from the field (loading, group work): aPR 0·60 (95% CI 0·40-0·89, p=0·012)  Carrying from the field (unloading, group work): aPR 0·51 (95% CI 0·35-0·75, p<0·001)  Piercing/Threading group work: aPR 0·63 (95% CI 0·35-1·14, p=0·124)  Curing in the burn (hanging up, group work): aPR 0·56 (95% CI 0·37-0·83, p=0·004)  Curing in the barn (climbing down, group work): aPR 0·56 (95% CI 0·37-0·83, p=0·004)  Bailing small size group work: aPR 0·70 (95% CI 0·47-1·04, p=0·074)  Bailing medium size group work: aPR 0·51 (95% CI 0·36-0·73, p<0·001)  Bailing large size group work: aPR 0·36 (95% CI 0·25-0·51, p<0·001)  Transportation loading group work aPR 0·45 (95% CI 0·30-0·68, p<0·001)  Transportation unloading group work: aPR 0·49 (95% CI 0·32-0·75, p=0·001)  Wrist Planting season-  Seedling 4-6h: aPR 0·77 (95% CI 0·45-1·32, p=0·342)  Seedling 1-3h: aPR 0·90 (95% CI 0·45-1·81, p=0·898)  Plowing using hand tractor: aPR 1·19 (95% CI 0·52-2·72, p=0·673)  Digging holes manual: aPR 0·55 (95% CI 0·15-2·00, p=0·363)  Planting 4-6h: aPR 0·60 (95% CI 0·34-1·05, p=0·075)  Planting 1-3h: aPR 0·53 (95% CI 0·26-1·10, p=0·089)  Crop maintenance spraying with backpack: aPR 0·82 (95% CI 0·47-1·42, p=0·479)  Topping the tobacco plant 4-6h: aPR 1·21 (95% CI 0·69-2·12, p=0·501)  Topping the tobacco plant 1-3h: aPR 0·13 (95% CI 0·02-0·97, p=0·047)  Wrist Harvesting season-  Carrying from the field (loading, group work): aPR 0·74 (95% CI 0·94-1·39, p=0·351)  Carrying from the field (unloading, group work): aPR 0·67 (95% CI 0·37-1·22, p=0·188)  Piercing/Threading group work: aPR 0·32 (95% CI 0·24-0·44, p<0·001)  Curing in the burn (hanging up, group work): aPR 1·21 (95% CI 0·64-2·30, p=0·553)  Curing in the barn (climbing down, group work): aPR 0·55 (95% CI 0·34-0·89, p=0·015)  Bailing small size group work: aPR 0·50 (95% CI 0·35-0·71, p<0·001)  Bailing medium size group work: aPR 0·43 (95% CI 0·30-0·63, p<0·001)  Bailing large size group work: aPR 0·46 (95% CI 0·27-0·78, p=0·004)  Transportation loading group work: aPR 0·39 (95% CI 0·23-0·67, p=0·001)  Transportation unloading group work: aPR 0·37 (95% CI 0·22-0·65, p<0·001)  Lower back Planting season-  Seedling 4-6h: aPR 0·63 (95% CI 0·41-0·97, p=0·035)  Seedling 1-3h: aPR 0·62 (95% CI 0·34-1·13, p=0·120)  Plowing using hand tractor: aPR 1·13 (95% CI 0·54-2·35, p=0·746)  Digging holes manual: aPR 0·71 (95% CI 0·24-2·14, p=0·544)  Planting 4-6h: aPR 0·96 (95% CI 0·58-1·58, p=0·870)  Planting 1-3h: aPR 1·08 (95% CI 0·61-1·91, p=0·783)  Crop maintenance spraying with backpack: aPR 0·50(95% CI 0·32-0·78, p=0·002)  Topping the tobacco plant 4-6h: aPR 1·08 (95% CI 0·71-1·66, p=0·718)  Topping the tobacco plant 1-3h: aPR 0·40 (95% CI 0·16-0·98, p=0·045)  Lower back Harvesting season-  Carrying from the field (loading, group work): aPR 0·88 (95% CI 0·63-1·22, p=0·434)  Carrying from the field (unloading, group work): aPR 0·81 (95% CI 0·58-1·12, p=0·201)  Piercing/Threading group work: aPR 0·61 (95% CI 0·43-0·88, p=0·008)  Curing in the burn (hanging up, group work): aPR 1·06 (95% CI 0·77-1·46, p=0·705)  Curing in the barn (climbing down, group work): aPR 0·76 (95% CI 0·56-1·03, p=0·080)  Bailing small size group work: aPR 0·85 (95% CI 0·63-1·16, p=0·311)  Bailing medium size group work: aPR 0·75 (95% CI 0·58-0·98, p=0·033)  Bailing large size group work: aPR 0·53 (95% CI 0·40-0·71, p<0·001)  Transportation loading group work: aPR 0·72(95% CI 0·50-1·04, p=0·082)  Transportation unloading group work: aPR 0·72 (95% CI 0·50-1·03, p=0·074)  Knee Planting season-  Seedling 4-6h: aPR 0·75 (95% CI 0·51-1·11, p=0·753)  Seedling 1-3h: aPR 1·04 (95% CI 0·67-1·63, p=0·857)  Plowing using hand tractor: aPR 2·29 (95% CI 1·19-4·39, p=0·013)  Digging holes manual: aPR 1·31 (95% CI 0·62-2·78, p=0·474)  Planting 4-6h: aPR 0·93 (95% CI 0·62-1·42, p=0·744)  Planting 1-3h: aPR 0·86 (95% CI 0·52-1·42, p=0·555)  Crop maintenance spraying with backpack: aPR 0·61 (95% CI 0·41-1·17, p=0·012)  Topping the tobacco plant 4-6h: aPR 0·79 (95% CI 0·54-1·17, p=0·244)  Topping the tobacco plant 1-3h: aPR 0·51 (95% CI 0·26-0·99, p=0·048)  Knee Harvesting season-  Carrying from the field (loading, group work): aPR 0·79 (95% CI 0·53-1·20, p=0·273)  Carrying from the field (unloading, group work): aPR 0·60 (95% CI 0·40-0·88, p=0·009)  Piercing/Threading group work: aPR 0·71 (95% CI 0·43-1·16, p=0·172)  Curing in the burn (hanging up, group work): aPR 0·68 (95% CI 0·46-1·01, p=0·053)  Curing in the barn (climbing down, group work): aPR 0·57 (95% CI 0·39-0·84, p=0·004)  Bailing small size group work: aPR 0·76 (95% CI 0·55-1·05, p=0·099)  Bailing medium size group work: aPR 0·83 (95% CI 0·58-1·18, p=0·295)  Bailing large size group work: aPR 0·31 (95% CI 0·21-0·44, p<0·001)  Transportation loading group work: aPR 0·54 (95% CI 0·36-0·83 , p=0·005)  Transportation unloading group work: aPR 0·61 (95% CI 0·38-0·97, p=0·036) |
| Kumar 1999; India | 28 (56%) of tractor driving farmers (TDF) had back symptoms, compared with 16 (32%) of non-tractor driving farmers (NTDF) (p=0·015).  7 (14%) of TDF were found to have abnormal right knee clinical examinations compared to 0 (0%) of no NTDF (p=0·021).  7 (14%) of TDF were found to have abnormal left knee clinical examinations compared to 0 (0%) of NTDF (p=0·021) |
| Liu 2012; China | Multivariate logistic regression reported: (no p value reported)  Sex: NS  Education: NS  Age: 15-24y ref, 25-34 OR 2·12 (1·36-3·30), 35-44 OR 3·29 (2·14-5·07), 45-54 OR 4·16 (2·67-6·48), 55-64 OR 4·00 (2·52-6·35), 65-74 OR 4·97 (2·82-8·75), 75-84 OR 5·43 (2·32-12·73)  Perceived stress:  Regularly: OR 2·51 (2·00-3·16)  Main farm activities: NS  Smoker: NS  Drinker: NS |
| Manothum; 2018; Thailand | Age is significantly associated with pain in the neck, upper back, lower back, upper arm, elbow and hand (p<0·01) (no other data available).  Experience was associated with pain in the shoulder, lower arm, hand, calf,and foot (p<0·01) (no raw data presented). |
| McNeill; 1998; Ghana | Activities specifically attributed to pain are:  Weeding 31%, Mound making 16%, Bed making 3%, Clearing 2%, Stumping 28%, Planting 11%,  Harvesting 4% |
| Meksawi; 2012; Thailand | Adjusted ORs (adjusted for age, sex, BMI, underlying disease):  Tapping level (ref waist-eye): Below waist OR 3·57 (5·77, 31·9) p=<0·001  Above eye and below waist OR 5·22 (2·06,13·23) p=<0·001  Frequency of trunk twisting (ref mild): High OR 2·07 (1·07, 3·99) p=0·03  Frequency of bending (ref mild): High OR 2·48 (1·2,5·11) p=0·01  Frequency of lifting (ref never): High OR 5·27 (1·26,21·99) p=0·02  Frequency of trunk extension (ref never): High OR 1·2 (1·00,1·35) p=0·02  Work fatigue (ref never): Mild OR 3·80 (1·68,8·59) p=0·001, Moderate OR 2·87 (1·25-6·6) p=0·01, High OR 3·41 (1·22, 9·51) p=0·02  Social support: (ref high): Mild OR 3·29 (1·44,7·52) p=0·005  Educational level (ref high school): Primary school OR 2·40 (1·04-5·53) p=0·04  Average income per month) ref 20,000 bhat): <20,000 bhat OR 2·08 (1·07-4·05) p =0·03 |
| Meucci; 2015; Brazil | Multivariate adjusted analysis:  Age (18-29ref): 30-39 PR 3·00 (1·70-5·28), 40-49 PR 4·99 (2·93-8·50), 50+y PR 5·14 (3·04-8·71), P<0·001  Gender: NS  Amount of tobacco produced (kg): NS  Livestock rearing no· species: None ref, 1: PR 0·92 (0·63-1·33), 2+: PR 1·65 (1·14-2·38) p=0·02   smoking: NS  Climbing high into curing barn: NS  Bottom leaf harvesting: NS  Tasks requiring heavy physical exertion (no ref): PR 2·00 (1·43-2·79) p=<0·001  Working in awkward postures: PR 1·36 (1·02-1·82) p=0·03  Green Tobacco Sickness: (no ref): PR 1·63 (1·18-2·25) p=0·003  Pesticide poisoning (life) (no ref): PR 2·37 (1·70-3·32) p=<0·001  Minor psychiatric disorders (no ref): PR 2·55 (1·88-3·47) p=<0·001 |
| Milani; 2012; Brazil | Univariate analyses of Work Ability Index (WAI) and 12m pain reports:  low WAI scores (<37) associated with any pain site (p=0·02), shoulders (p=0·02), wrists/hands (p=0·01); other associations p>0·05· |
| Momeni 2020, Iran | Modelling of the association between the potential risk factors and MSD symptoms by region using multiple logistic regression (n=1501)  Neck:  Age: OR 1·017 (95% CI 1·01-1·02, p<0·001)  Posture: OR 1·62 (95% CI 1·03-2·56, p=0·035)  Shoulder:  Age: OR 1·53 (95% CI 1·15-2·10, p=0·002)  Elbows:  Age: OR 1·68 (95% CI 1·15-2·44, p=0·006)  Wrists/hands:  Handedness: OR 1·72 (95% CI 1·25-2·38, p=0·001)  posture: OR 1·56 (95% CI 1·01-2·42, p=0·045)  Upper back:  Age: OR 1·31 (95% CI 1·12-2·05, p=0·008)  Body mass: OR 1·71 (95% CI 1·35-2·25, p<0·001)  Lower back:  Age: OR 1·30 (95% CI 1·10-1·75, p=0·011)  Body mass: OR 1·52 (95% CI 1·05-2·28, p=0·020)  Posture: OR 1·89 (95% CI 1·16-3·08, p=0·010)  Thighs:  Age: OR 1·032 (95% CI 1·02-1·04, p<0·001)  Education level: OR 1·37 (95% CI 1·11-2·21, p=0·003)  Knees:  Age: OR 1·022 (95% CI 1·01-1·48, p<0·001)  Body mass: OR 1·34 (95% CI 1·02-1·78, p=0·031)  Legs/feet:  Age: OR 1·025 (95% CI 1·01-1·03, p<0·001)  Body mass: OR 1·61 (95% CI 1·24-2·11, p=0·001) |
| Mushayi; 2014; Zimbabwe | the frequently performed and commonly reported activities that were identified as high exposure to MSK disorders included standing for long periods  (73·3%), walking for long periods (73·3%), working for prolonged periods in the same posture (70%), performing repetitive forceful tasks (63·3%), working prolonged periods squatting or kneeling (56·7%)· Performing overhead activities, walking with the back bent, and pulling or pushing heavy objects were significantly associated with pain (p<0·05)· |
| Naidoo 2009; Africa | Univariate analysis adjusted for age and number of years worked found women aged over 40 more likely to report chronic pain in upper extremities (PR: 1·3 95% CI 1·1-1·4), lower extremities (PR 1·7, 1·5-2·0) and back (PR 1·3, 1·1-1·6) than their younger counterparts·  Working for more than 10 years was associated with more chronic pain in upper extremities (PR 1·3, 1·1-1·4), lower extremities (PR 1·4 (1·1-1·6) and back (PR 1·5 (1·2-1·7))· Perception of physical exertion at work was associated with all three chronic pain patterns: upper extremity pain PR 1·2 (1·1-1·5), back pain PR 1·3 (1·1-1·6), lower extremity pain PR 1·3 (1·1-1·6) all p<0·05·  Multivariate analyses:  Age (40+y): chronic upper extremity pain PR 1·2 (1·1-1·4), chronic lower extremity pain PR 1·9 (1·6-2·4), chronic back pain PR 1·3 (1·1-1·6)  Frequent squatting or kneeling: chronic upper extremity pain PR 1·7 (1·4-2·1), chronic lower extremity pain PR 1·4 (1·2-1·6), chronic back pain PR 1·7 (1·4-2·1)  Working with hands above shoulder height: chronic upper extremity pain PR 1·8 (1·4-2·1), chronic lower extremity pain PR 1·3 (1·1-1·6), chronic back pain PR 1·7 (1·4-2·1);  Lifting or carrying loads over 5kg: chronic upper extremity pain 2·5 (1·9-3·2), chronic lower extremity pain PR 1·8 (1·4-2·2), chronic back pain 2·3 (1·7-3·1)  Working in awkward positions: chronic upper extremity pain – not significant, chronic lower extremity pain PR 0·7 (0·5-0·8),chronic back pain PR 0·5 (0·4-0·6)  All reported are p<0·05 |
| Nawi, 2016; Malaysia | Body part pain based on work units within palm oil plantation (no reported CIs or p values):  Cutter / Frond Stacker / loose fruit collector / badang driver / MTG truck driver (n, (%))  Neck: 32 (36·4%) / 5 (5·7%) / 19 (21·6%) / 2 (2·3%) / 7 (8·0%); Shoulder right 30 (34·1%) / 9 (10·2%) / 21 (23·9%) / 5 (5·7%) / 6 (6·8%); Shoulder left 29 (33·0%) / 9 (10·2) /  20% (22·7) / 5 (5·7%) / 7 (8·0%); Elbow right 15 (17·0%) / 3 (3·4%) / 16 (18·2%)/ 2 (2·3%)/ 3 (3·4%); Elbow left12 (13·6%) / 3 (3·4%)/ 16 (18·2%) / 2 (2·3%) / 3 (3·4%); Upper back 31 (35·2%) / 9 (10·2%) / 24 (27·3%)/ 5 (5·7%) / 6 (6·8%); Lower back 33 (37·5%) / 10 (11·4%) / 32 (36·4%) / 5 (5·7%) / 7 (8·0%); Finger right: 13 (14·8%) / 2 (2·3%) / 16 (18·2%) / 1 (1·1%) / 3 (3·4%); Finger left 12 (13·6%) / 2 (2·3%) / 16 (18·2%) / 1 (1·1%) / 3 (3·4%); Upper arm right 24 (27·3%) / 5 (5·7%) / 14 (15·9%) / 3 (3·5%) / 5 (5·7%) ; Upper arm left 22 (25·0%) / 4 (4·5%) /14 (15·9%) / 4 (4·5%) / 5 (5·7%) ; Lower arm right 16 (18·2%) / 2 (2·3%) / 15 (17·0%) / 2 (2·3%) / 5 (5·7%) ; Lower arm left 15 (17·0%) / 2 (2·3%) / 16 (18·2%) / 2 (2·3%) / 3 (3·4%) ; Wrist 17 (19·3%) / 3 (3·4%) / 16 (18·2%) / 2 (2·3%) / 3 (3·4%) ; Ankle right 10 (11·4%) / 2 (2·3%) / 16 (18·2%) / 1 (1·1%) / 5 (5·7%); Ankle left 10 (11·4%) / 2 (2·3%) / 16 (18·2%) / 1 (1·1%) / 5 (5·7%); Buttock right 25 (28·4%) / 7 (8·0%) / 25 (28·4%) / 4 (4·5%) / 7 (8·0%); Buttock left 24 (27·3%) / 7 (8·0%) / 25 (28·4%) / 4 (4·5%) / 7 (8·0%);  Knee right 14 (15·9%) / 4 (4·5%) / 24 (27·3%) / 2 (2·3%) / 5 (5·7%) ; Knee left 14 (15·9%) / 4 (4·5%) / 25 (28·4%) / 2 (2·3%) / 5 (5·7%); Calf right 23 (26·1%) / 4 (4·5%) / 29 (33·0%) / 2 (2·3%) / 5 (5·7%) ; Calf left 23 (26·1%) / 4 (4·5%) / 29 (33·0%) / 2 (2·3%) / 5 (5·7%); Foot right 11 (12·5%) / 3 (3·4%) / 10 (11·4%) / 1  (1·1%) / 4 (4·5%); Foot left 12 (13·6%) / 3 (3·4%) ; 10 (11·4%) ; 1 (1·1%) ; 4 (4·5%); Thigh right 13 (14·8%) / 4 (4·5%) / 21 (23·9%) / 3 (3·4%) / 5 (5·7%); Thigh left 13 (14·8%) / 4 (4·5%) / 21 (23·9%) / 3 (3·4%) / 5 (5·7%) |
| Ng 2015; Malaysia | Multivariate analysis for MSDs for FFB cutters in the past 12 months (with p<0·05):  Education: secondary education (OR 9·412 (2·139-41·406)  For neck disorders in the last 7 days for FFB cutters:  Daily resting duration of 51 minutes or more (OR 3·705 (1·046-13·121) compared to reference 50 minutes or less;  For lower back disorders in the last 7 days for FFB cutters:  Increasing BMI OR 0·837 (0·717-0·975);  Duration of employment longer than 20 months OR 0·484 (0·244-0·961) (reference is employment duration <=20 months);  Posture after cutting FFB) OR 3·31 (1·252-8·793) reference is before cutting FFB·  Associations with 12m MSDs for collectors:  Neck: daily working duration in minutes (OR 0·993 (0·998-0·998); working overtime (OR 2·907 (1·187-7·121); fishing (OR 3·219 (1·533-6·757);  Shoulder: daily working duration (minutes): OR 2·514 (1·207-5·237)  Associations with 7d MSDs in collectors:  Shoulder: daily working duration (minutes) OR 0·994 (0·988-0·999); daily resting duration (reference 50 minutes): OR 2·857 (1·083-7·539)·  Neck: walking with restricted posture OR 2·974 (1·202-7·361)  Lower back: collecting loose fruits OR 4·139 (1·502-11·405);  Knee: walking with restricted posture OR 4·621 (1·046-20·424);  Total MSDs: collecting loose fruits OR 5·571 (1·548-20·057) |
| Omokhodion 2002; Nigeria | 40% of farmers thought their LBP was due to heavy physical work; 46% of farmers thought their LBP was due to bending (no significance testing was reported) |
| Omokhodion 2004; Nigeria | Of farmers, 82% of men and 100% women reported LBP; no CI or p-values were reported |
| Phajan 2014; North-Eastern Thailand | In bivariate analyses, extending or twisting wrist repeatedly for more than 2 hours per day OR 2·70 (1·29-5·65) p=0·008; also repetitive motions (OR 2·38 (1·37-4·14, p=0·02), prolonged standing (OR 3·67 (1·88-7·17, p<0·001), hand squeezing or exertion (OR 2·39 (1·32-4·34, p=0·004), awkward postures (OR 3·28 (1·82-5·91, p<0·001)), twisting torso (OR 2·27 (1·19-4·32, p0·012)), forceful exertions e·g· heaving lifting (OR 3·52 (2·12-8·97, p<0·001), and stress about future income (OR 1·88 (1·09-3·22, p0·023))·  Multivariate adjusted analysis (for age, sex, BMI, smoking, exercise):  Repetitive motions OR 1·90 (1·05-3·43); awkward postures (OR 1·95 (1·01-3·77); forceful exertions (OR 2·78 (1·54-5·02); stress over future income (OR 1·80 (1·02-3·16) |
| PinargoteCedeno 2021, Ecuador | Adjusted OR for Shoulder in farmer: aOR 9·78 (95% CI 4·41-24·65, p value not specified)  Adjusted OR for lower back in farmer: aOR 1·77 (95% CI 0·8-3·7, p value not specified) |
| Razavi, 2014; Iran | Raised BMI was significantly associated with pain (no p-value reported)  More exercise was associated with reduced reports of pain (no p-value reported)  Increasing age was associated with prevalence of MSDs (p<0·05)  Working for more than 20 years was associated with back (p=0·008), wrist (p=0·01), upper arm (p=0·03), foot (p=0·03) and neck (p=0·04)· |
| Reddy 2012, India | Neck pain was associated with increasing age in bivariate analysis (p<0·001) |
| Rocha 2014; Brazil | Activities and pain locations that were statistically significant (p<0·05) are:  Head and production management, lumbar/sacral spine and soil preparation, calves and culture treatment, feet and production planning, hands and production planning, feet and production management  Pain reports were statistically higher in those reporting stress (n=98, 37·8%, p=<0·05), anxiety (n=94, 36·0%, p<0·05), disorders of sleep-wake cycle (n=70, 26·9%, p=0·025) |
| Shan 2012; Malaysia | Multivariate logistic regression model for neck pain:  Age (referent is age 59y +)  39-48y OR 3·92 (1·61-9·58), p=0·003;  49-58y OR 1·80 (0·88-3·70), NS  Static postures (no pain is reference):  Yes OR 1·86 (1·11 -3·14), p=0·021  Awkward posture (no pain is reference):  Yes OR 2·23 (1·29-3·86), p=0·004;  Neck flexion or rotation (no pain is referent):  Yes OR 9·52 (5·55-16·32), p=0·001 |
| Singh 2022, India | Normal activities reported disrupted due to pain by n=99 (65·13%). 4 farmers reported medication relief for low back pain |
| Taechasubamorn 2011; Thailand | Sex:  Lifetime LBP: male 101 (46·1%), female 118 (53·9%), p=0·100;  Within 12 months LBP: male 70 (44·0%), female 89 (56·0%), p=0·071;  Point prevalence LBP: male 60 (43·2%), female 79 (56·8%), p=0·064;  Age: NS, though highest prevalence was found in youngest group (66·7%).  Rice farmers experienced increased LBP from slouched sitting (56·2%), forward bending (70·8%), lifting (83·2%) and decreased LBP from straight sitting (41·6%) and back extension in standing (58·6%) (no measures of significance reported). |
| Tella 2013; Nigeria | males had significantly higher 12 month prevalence than females (78·4% male, 21·6% female (p<0·05)), and point prevalence (no absolute values reported, p<0·001). |
| Thetkathuek 2017; Thailand | Women were at risk of hip and thigh pain (aOR 1·85 (1·29-2·64) and wrist and hand pain compared with men (aOR 1·56 (1·03-2·36).  Men aged 40-49 years (aOR 8·78 (1·04-74·18) and >50years (aOR 7·63 (7·59-98·78) were at increased risk of knee pain when compared with those < 20y  Men working >10 years had higher risk of neck pain (aOR 1·66 (1·90-14·5) compared to those < 1y  Men working on plantation area >39 hectars had increased risk of pain in neck, wrist, upper and lower back, knee and foot compared to workers on <20 acres (aOR 2·45 (1·35-4·45 / OR3·57 (1·68-7·59) / 2·50 (1·44-4·33) / 3·53 (2·03-6·11) / 2·96 (1·44-6·0) / 3·94 (1·92-8·09)  Men whose tasks required raising of arms above shoulder height increased neck pain risk (aOR 1·68 (1·08-2·61)  Women working 6-10 years (aOR 5·92 (1·02-34·3) and > 10 years (aOR 8·13 (1·04-63·74) had increased risk of lower back pain compared to those working for < 1 year  Women working in plantation > 39 acres had increased risk of pain in the neck, elbow, wrist, upper and lower back, knee, foot compared to those working in plantation areas < 20 acres (aOR 3·03 (1·58-5·80) / 3·50 (1·20-10·19) / 4·00 (1·93-8·3) / 2·82 (1·50-5·30) / 2·83 (1·50-5·36) / 4·69 (2·21-9·94) / 3·16 (1·44-6·94) |
| Udom 2016; Thailand | Increasing BMI (aOR1·05 (1·00-1·11, p=0·048), primary school education (referent is post-secondary school, aOR 2·45 (1·13-5·32, p=0·013), exposure to pesticides (referent is no; aOR 1·63 (1·04-2·55, p=0·044), tapping level below knee (reference is above eye; aOR 2·64 (1·02-6·85, p=0·049) were significantly associated with LBP after adjusting for age, sex, BMI, education, underyling disease, exposure to pesticides, physical activity, tapping level and stress level |
| Vasanth 2015; India | The mean age of those with pain was 6·59 years more than those with no pain (no pain mean age 40·25y, yes pain mean age 46·84y, p<0·01);  The mean years of employment was 1·38 years more among the workers with pain compared to workers without pain (no pain mean 3·31, yes pain mean 4·7, p<0·01)  Comorbidities associated with increase in MSDs (No comorbidities and pain 77·9%, comorbidities and pain 90·1%, p=0·021) |
| Worku 2000; South Africa | Intensive farm work, high gravidity, rural residence, heavy weight lifting, low literacy status, strenuous manual labour, no outreach health services, low income, firewood cooking method, breastfeeding all p<0·05 (no raw data or summary risk estimate presented) |
| Xie 2020; China | The group with high exposure to pesticides reported statistically significantly more pain, swelling and weakness in the joints of the hands of feet then the group with low exposure to pesticides (p=0·024) |

Supplementary Information 5: table 4· Associated burdens of MSDs as reported in prevalence studies

| **Study Author, Country** | **Associated Burdens of MSK Disorders** |
| --- | --- |
| CruzeiroSzortyka 2021; Brazil | low back pain and suicidal ideation unadjusted prevalence ratio 4.17 (95% CI 2.43-7.16) (P<0.001), suicide attempt unadjusted prevalence ratio 4.32 (95% CI 1.93-9.68) p<0.001. Low back pain and suicidal ideation adjusted prevalence ratio 1.83 (95% CI 1.04-3.21) p=0.037 |
| Dianat 2020; Iran | 12-month prevalence of work interference due to MSS (all farmers): Neck n=197 (52.3%), shoulders n =59 (15.6%), elbows n=28 (7.4%), hands/wrist n=62 (16.4%), upper back n=132 (35.0%), low back n=220 (58.4%) hips/thighs/buttocks n=58 (15.4%), knees n=171 (45.4%), ankles/feet n=46 (12.2%), any region n=272 (72.1%). male farmers: Neck n=68 (45.6%), shoulders n =13 (8.7%), elbows n=6 (4.0%), hands/wrist n=16 (10.7%), upper back n=44 (29.5%), low back n=78 (29.5%) hips/thighs/buttocks n=10 (6.7%), knees n=56 (37.6%), ankles/feet n=13 (8.7%), any region n=92 (61.7%). female farmers: Neck n=129 (56.6%), shoulders n =46 (20.2%), elbows n=22 (9.6%), hands/wrist n=46 (20.2%), upper back n=88 (38.6%), low back n=142 (62.3%) hips/thighs/buttocks n=48 (21.1%), knees n=115 (50.4%), ankles/feet n=33 (14.5%), any region n=180 (78.9%). |
| Fabunmi 2005; Nigeria | 188 (51.9%) of respondents indicated LBP hindered them from farm work, 124 (34.3%) were disturbed from walking, 87 (24.0%) were not able to enjoy hobbies, 157 (43.3%) had sleep disturbed. |
| Kairi 2022; Bangladesh | Hospitalisation n=56 (16.1%). Consultation with doctor/physiotherapist n=280 (80.9%). Medication due to MSS in last 12 months: n=279 (80.6%). Sick leave in the past 12 months: n=271 (78.3%). Change of jobs: n=40 (11.6%). Reduced work activity in the past 12 months: n=269 (77.7%). Reduced leisure activity in the past 12 months: n=265 (76.6%) |
| Karimi 202; Iran | Interference with work   Not at all:  Pre-intervention: neck n=14 (29.2%), shoulder right n=26 (54.2%), shoulder left n=17 (35.4%), upper back n=13 (27.0%), upper arm right n=1 (2.1%), upper arm left n=1 (2.1%), lower back n=5 (10.4%), forearm right n=9 (18.5%), forearm left n=9 (18.5%), wrist right n=4 (8.3%), wrist left n=4 (8.3%), hip/buttocks n=3 (6.3%), thigh right n=5 (10.4%), thigh left n=5 (10.4%), knee right n=4 (8.3%), knee left n=4 (8.3%), lower leg right n=3 (6.3%), lower leg left n=3 (6.3%), foot right unavailable and foot left unavailable.   Post-intervention: neck n=11 (22.9%), shoulder right n=15 (31.2%), shoulder left n=15 (31.2%), upper back n=8 (16.7%), upper arm right unavailable, upper arm left unavailable, lower back n=11 (22.9%), forearm right n=4 (8.3%), forearm left n=5 (10.4%), wrist right n=2 (4.1%), wrist left n=2 (4.1%), hip/buttocks n=2 (4.1%), thigh right n=4 (8.3%), thigh left n=4 (8.3%), knee right n=4 (8.3%), knee left n=5 (10.4%), lower leg right n=6 (12.5%), lower leg left n=6 (12.5%), foot right unavailable and foot left unavailable.   Slightly interfered:  Pre-intervention: neck n=4 (8.3%), shoulder right n=3 (6.3%), shoulder left n=3 (6.3%), upper back unavailable, upper arm right unavailable, upper arm left unavailable, lower back n=16 (33.3%), forearm right n=3 (6.3%), forearm left n=3 (6.3%), wrist right n=2 (4.1%), wrist left n=2 (4.1%), hip/buttocks unavailable, thigh right n=2 (4.1%), thigh left n=2 (4.1%), knee right n=4 (8.3%), knee left n=4 (8.3%), lower leg right n=7 (14.6%), lower leg left n=7 (14.6%), foot right unavailable and foot left unavailable.   Post-intervention: neck n=1 (2.1%), shoulder right n=2 (4.1%), shoulder left n=1 (2.1%), upper back n=1 (2.1%), upper arm right unavailable, upper arm left unavailable, lower back n=7 (14.6%), forearm right unavailable, forearm left unavailable, wrist right unavailable, wrist left unavailable, hip/buttocks unavailable, thigh right unavailable, thigh left unavailable, knee right n=1 (2.1%), knee left n=1 (2.1%), lower leg right n=2 (4.1%), lower leg left n=2 (4.1%), foot right unavailable and foot left unavailable.   Substantially interfered:  Pre-intervention: neck unavailable, shoulder right n=2 (4.1%), shoulder left n=2 (4.1%), upper back unavailable, upper arm right unavailable, upper arm left unavailable, lower back n=12 (25.0%), forearm right unavailable, forearm left unavailable, wrist right unavailable, wrist left unavailable, hip/buttocks unavailable, thigh right unavailable, thigh left unavailable, knee right n=5 (10.4%), knee left n=5 (10.4%), lower leg right n=1 (2.1%), lower leg left n=1 (2.1%), foot right unavailable and foot left unavailable.   Post-intervention: neck unavailable, shoulder right unavailable, shoulder left unavailable, upper back unavailable, upper arm right unavailable, upper arm left unavailable, lower back n=2 (4.1%), forearm right unavailable, forearm left unavailable, wrist right unavailable, wrist left unavailable, hip/buttocks unavailable, thigh right unavailable, thigh left unavailable, knee right n=1 (2.1%), knee left n=1 (2.1%), lower leg right n=1 (2.1%), lower leg left n=1 (2.1%), foot right unavailable and foot left unavailable. |
| Kaur 2022 | MSK discomfort that prevent them from carrying out their activities during the previous 12 months reported as bar graph which does not have any raw data (numerator/denominator). |
| Liu 2012; China | 66.0% of those with back pain reported that it affected work quantity and quality. |
| McNeill 1998; Ghana | Back pain is associated with, on average, 19 days lost from work |
| Mushayi; 2014; Zimbabwe | 56.5% of the workers who reported MSK complaints also mentioned these problems affected their day to day activities. |
| Omokhodion 2002; Nigeria | health seeking - 44% of farmers reported consulting health care personnel |
| PinargoteCedeno 2021, Ecuador | Farmers n=106 12 month interference with work: Neck n=8 (7.54%), shoulder n=18 (16.98%)*, thoracic spine n=8 (7.54%), lumbar spine n=18 (16.98%) *denotes significant difference between farmers and administrative staff |
| Singh 2022, India | Normal activities reported disrupted due to pain by n=99 (65.13%). 4 farmers reported medication relief for low back pain. |
| Tella 2013; Nigeria | Difficulty carrying out farm work due to LBP (54.1%), disturbed sleep (17.1%). Prevention in formerly practiced activities e.g. sex (13.7%), attending parties (8.9%), hunting (4.3%), marketing (8%). Absence from farm due to LBP (57.8%). |
| Vasanth 2015; India | A majority of the workers, i.e., 194 (99.5%) had seen the doctor for neck pain and lower back pain; among them 15 (7.4%) were hospitalized for lower back pain. Among those with shoulder pain, 8 (4.1%) had to change jobs. Duration of pain for less than 1 month was more in the shoulders, i.e., in 31 (15.5%) workers and pain for more than 1 month was more in the lower back and neck, i.e., in 100 (51.3%) workers. |

Supplementary Information 6: Table 5· Quality appraisal of selected studies

| **Author, year** | **1** | **2** | **3** | **4** | **5** | **6** | **7** | **8** | **9** | **10** | **11** | **Overall score** |
| --- | --- | --- | --- | --- | --- | --- | --- | --- | --- | --- | --- | --- |
| Barerro 2006 | Low | Low | Low | Low | Low | High | Low | Low | Low | Low |  | Moderate |
| Barerro 2012 | Low | Low | Low | Low | Low | Low | Low | Low | Low | Low |  | Low |
| Basher 2015 | High | High | High | High | High | High | High | High | Low | High | high risk due to limited information in abstract | High |
| Bhandari 2018 | High | High | Low | High | Low | High | High | High | High | High |  | High |
| Bihari 2011 | High | Low | Low | Low | Low | Low | High | Low | Low | High |  | Low |
| Birabi 2012 | High | Low | Low | High | Low | Low | Low | Low | Low | Low | No record of non-response, and 'researchers corrected questionnaires' | Moderate |
| Caminiti Tejada 2011 | High | High | High | High | Low | High | High | Low | Low | Low | overall moderate as data was limited as it was a secondary analysis so no info on some areas e·g· non-responders | Moderate |
| Carvalho 2020 | High | Low | Low | Low | Low | Low | Low | Low | Low | Low |  | Low |
| Chokprasit 2022 | High | Low | Low | Low | Low | Low | Low | Low | High | Low |  | Low |
| CuzeiroSzortyka 2021 | High | Low | Low | Low | Low | Low | Low | Low | Low | Low |  | Low |
| Das 2013 | High | High | High | High | High | High | Low | Low | High | Low |  | Moderate |
| Das 2015 | High | High | Low | High | Low | Low | Low | Low | High | High |  | Moderate |
| Diallo 2020 | High | High | High | High | Low | High | High | Low | High | High | Insufficient information provided to allow reproducibility | HIgh |
| Dianat 2020 | High | Low | Low | High | Low | Low | Low | Low | Low | Low |  | Low |
| Emami 1998 | Low | Low | Low | High | Low | Low | Low | Low | Low | Low | low risk as large sample and likely to be representative | Low |
| Fabunmi 2005 | High | Low | Low | Low | Low | High | High | Low | Low | Low | no definition of LBP, this may change estimates | Moderate |
| Fassa 2020 | High | Low | Low | Low | Low | Low | Low | Low | Low | Low |  | Low |
| Gupta 2013 | Low | Low | Low | High | Low | Low | Low | Low | Low | Low | overall moderate as there were a lot of exclusions and no info on non-responders | Moderate |
| Hasan 2022 | High | High | High | High | Low | High | High | Low | High | Low | Insufficient information provided to allow reproducibility | High |
| Hongsibsong 2017 | High | High | Low | Low | Low | High | High | Low | High | Low |  | Moderate |
| Jain 2018 | High | High | High | High | Low | High | High | Low | Low | Low | high risk as no information on study sample frame or derivation | High |
| Kar 2007 | High | High | Low | High | Low | High | High | Low | Low | High | likely similar result if repeated but cannot overlook potential biases | Moderate |
| Kaewdok 2021 | HIgh | High | High | High | Low | Low | Low | Low | Low | High | No information on response rate | Moderate |
| Kairi 2022 | High | High | High | High | Low | Low | Low | Low | Low | Low | Only 1 tea plantation included | Moderate |
| Karimi 2020 | High | High | High | Low | Low | Low | Low | Low | Low | Low |  | Moderate |
| Kaur 2022 | High | High | High | Low | Low | Low | Low | Low | Low | Low | quota sampling is an accepted methodology though not randomised | Moderate |
| Keawduangdee 2015 | High | High | Low | High | Low | Low | Low | Low | Low | Low |  | Moderate |
| Kongtawelert 2022 | Low | Low | Low | High | Low | Low | Low | Low | Low | High | Risk of recall bias | Moderate |
| Kumar 1999 | High | Low | High | High | Low | High | High | Low | Low | Low | A further study is likely to yield similar results 50 TDFs selected from 129 households | Moderate |
| Liu 2012 | High | Low | Low | Low | Low | High | Low | Low | Low | Low |  | Low |
| Manothum 2018 | High | High | High | High | Low | High | Low | Low | High | Low |  | Moderate |
| McNeill 1998 | Low | Low | High | High | Low | High | High | Low | Low | Low | not much information in the methodology section to draw confident conclusions so will have to be considered high risk | High |
| Meksawi 2012 | Low | Low | Low | Low | Low | Low | Low | Low | Low | Low | Large sample and representative, no info on response rate | Moderate |
| Meucci 2015 | High | Low | Low | High | Low | Low | Low | Low | Low | High | no absolute numbers presented | Moderate |
| Milani 2012 | High | High | High | Low | Low | Low | Low | Low | Low | Low | moderate, little information on representativeness and non-response, but standardised survey used· | Moderate |
| Momeni 2020 | High | High | Low | High | Low | Low | Low | High | Low | Low |  | Moderate |
| Moreira 2015 | Low | Low | Low | High | Low | High | High | Low | High | Low | not much information on methodology, but given national survey likely to be low risk, although can't judge as no information | Moderate |
| Mushayi 2014 | High | High | High | High | Low | Low | Low | Low | Low | Low | moderate because a reliable measure was used but no information on non-responders and small sample from only one area | Moderate |
| Naeini 2020 | High | High | High | High | Low | Low | Low | Low | Low | Low | moderate because although tool used is valid, problem is lack of information about the population, including NRs | Moderate |
| Naidoo 2009 | Low | Low | Low | Low | Low | High | Low | Low | Low | Low |  | Low |
| Nawi 2016 | High | Low | Low | Low | Low | Low | Low | Low | Low | Low |  | Low |
| Ng 2014 | High | High | Low | Low | Low | Low | Low | Low | Low | Low |  | Low |
| Ng 2015 | High | High | High | High | Low | High | Low | Low | Low | Low |  | Moderate |
| Omokhodion 2002 | Low | Low | Low | High | Low | Low | Low | Low | Low | Low | low, unlikely that another study would find a drastically different result | Low |
| Omokhodion 2004 | High | Low | Low | Low | Low | Low | Low | Low | Low | Low |  | Low |
| Phajan 2014 | High | Low | Low | High | Low | Low | Low | Low | Low | Low |  | Moderate |
| PinargoteCedeno 2021 | HIgh | High | Low | High | Low | Low | Low | Low | Low | Low |  | Moderate |
| Razavi 2014 | High | High | Low | High | Low | Low | Low | Low | Low | Low | moderate as large sample, but a lot of unknowns | Moderate |
| Reddy 2012 | Low | Low | Low | High | Low | Low | Low | Low | Low | Low | low risk as real large sample | Low |
| Rocha 2014 | High | High | Low | Low | Low | High | High | Low | Low | Low | As good a study under difficult circumstances | Moderate |
| Rojas 2015 | Low | Low | Low | High | High | Low | Low | High | Low | Low | Large sample size in total, census sampling frame, low risk of bias despite one country with 50% response rate | Low |
| Sahu 2013 | High | Low | High | Low | Low | High | High | Low | High | High | scored medium as, despite limitations, it is unlikely that another study would find different results | Moderate |
| Shan 2012 | High | Low | Low | Low | Low | Low | Low | Low | Low | Low | comprehensive study, well executed | Low |
| Silva 2017 | High | High | High | High | High | High | Low | Low | High | Low | high as little information on methods | High |
| Simas 2020 | High | High | High | Low | Low | Low | Low | Low | Low | Low |  | Moderate |
| Singh 2022 | High | High | Low | Low | Low | Low | Low | Low | Low | Low |  | Moderate |
| Taechasubamorn 2011 | High | Low | Low | High | Low | Low | Low | Low | Low | Low | probably low, but given no response rate, moderate risk given | Moderate |
| Tella 2013 | High | High | High | High | Low | High | High | Low | Low | Low | study probably provides a good estimate of LBP prevalence, but lack of detail prevents low risk of bias | Moderate |
| Thetkathuek 2017 | High | High | High | High | Low | Low | Low | Low | Low | Low | Moderate due to missing information, though good sample size | Moderate |
| Udom 2016 | High | Low | Low | High | Low | Low | Low | Low | Low | Low | Moderate as no information on RR | Moderate |
| Vasanth 2015 | High | High | Low | High | Low | Low | Low | Low | Low | Low | overall moderate no info on study population and non-responders | Moderate |
| Worku 2000 | Low | Low | Low | High | Low | High | High | High | High | High | difficult to assess because rigorous survey methodology but no report on LBP measure, even in the original survey used to collect symptoms as cited in the references | Moderate |
| Xie 2020 | High | High | Low | High | Low | High | Low | Low | High | Low | difficult summary as relatively large sample but no information on the MSK outcome at all and have excluded 'right back pain' as this was under the cardiovascular disease heading | Moderate |
| Zhou 2022 | High | High | Low | Low | Low | High | High | Low | Low | Low |  | Moderate |
